# Codon stabilization coefficient as a metric to gain insights into mRNA stability and codon bias and their relationships with translation

**DOI:** 10.1093/nar/gkz033

**Published:** 2019-01-30

**Authors:** Rodolfo L Carneiro, Rodrigo D Requião, Silvana Rossetto, Tatiana Domitrovic, Fernando L Palhano

**Affiliations:** 1 Programa de Biologia Estrutural, Instituto de Bioquímica Médica Leopoldo de Meis, Universidade Federal do Rio de Janeiro, Rio de Janeiro, RJ 21941-902, Brazil; 2 Departamento de Ciência da Computação, Universidade Federal do Rio de Janeiro, Rio de Janeiro, RJ 21941-902, Brazil; 3 Departamento de Virologia, Instituto de Microbiologia Paulo de Góes, Universidade Federal do Rio de Janeiro, Rio de Janeiro, RJ 21941-902, Brazil

## Abstract

The codon stabilization coefficient (CSC) is derived from the correlation between each codon frequency in transcripts and mRNA half-life experimental data. In this work, we used this metric as a reference to compare previously published *Saccharomyces cerevisiae* mRNA half-life datasets and investigate how codon composition related to protein levels. We generated CSCs derived from nine studies. Four datasets produced similar CSCs, which also correlated with other independent parameters that reflected codon optimality, such as the tRNA abundance and ribosome residence time. By calculating the average CSC for each gene, we found that most mRNAs tended to have more non-optimal codons. Conversely, a high proportion of optimal codons was found for genes coding highly abundant proteins, including proteins that were only transiently overexpressed in response to stress conditions. We also used CSCs to identify and locate mRNA regions enriched in non-optimal codons. We found that these stretches were usually located close to the initiation codon and were sufficient to slow ribosome movement. However, in contrast to observations from reporter systems, we found no position-dependent effect on the mRNA half-life. These analyses underscore the value of CSCs in studies of mRNA stability and codon bias and their relationships with protein expression.

## INTRODUCTION

The interplay between mRNA synthesis and degradation determines the half-life of mRNA molecules and results in the final transcript levels found in a cell (1). Several features, such as the mRNA secondary structure, sequence, structural elements located within the 5′and 3′UTRs and transcript length, can affect mRNA stability (1–3). Moreover, mRNA codon composition is emerging as a strong factor that affects both RNA stability and translation efficiency (4). The concept of codon optimality or nonuniform codon translation efficiency was developed through the study of codon bias, which describes the disproportional frequency at which distinct synonymous codons are present in the genome (5). Codon optimality is a term that takes into account competition between tRNA supply and demand during translation and is an important determinant of codon bias (6). In several examined species, including *Saccharomyces cerevisiae*, the cellular tRNA concentrations are proportional to the tRNA gene copy numbers (7); therefore, the relative number of tRNA genes present in the genome and the efficiencies of different wobble interactions can be used to derive the tRNA adaptation index (tAI) (8,9). Optimal codons are decoded by high abundance tRNA species and are translated more efficiently than non-optimal codons. Therefore, the tAI reflects the efficiency of tRNA usage by the ribosome (9).

In addition to translation efficiency, mRNA codon composition seems to predict mRNA stability. Herrick *et al.* showed that the percentage of rare codons present in unstable mRNAs in yeast was significantly higher than that in stable mRNAs (10). Recent experiments in bacteria, yeast, and metazoans have indicated that codon optimality is a major determinant of mRNA stability (4).

Recently, Coller and colleagues reported a different codon optimality metric system that was derived from mRNA half-life data (11). The authors measured the half-lives of thousands of yeast genes and found that some codons were enriched in the most stable mRNAs, whereas other codons were enriched in the most unstable mRNAs. Based on Pearson's correlation between the frequency of occurrence of each codon in each mRNA and the mRNA half-lives, the authors created the codon occurrence to mRNA stability coefficient (CSC) (11). Direct comparison between the codon stabilization coefficient calculated by Coller *et al.* and the tRNA adaptive index (tAI) revealed good agreement between these scores (11), suggesting a direct effect of this adaptability index on the RNA half-life.

Here, we show that the CSC is a useful metric to investigate how the mRNA half-life relates to the protein translation efficiency. This type of analysis has been complicated by the notable lack of reproducibility of genome-wide mRNA half-life experimental measurements. Comparisons between different datasets available in the literature have yielded poor correlations, often classifying the same mRNA molecule as both stable and unstable. This issue makes the identification of stability sequence motifs and the global analysis of transcription and translation dynamics problematic tasks (12,13). Calculation of CSCs from nine different data sets allowed us to find the most similar datasets and to identify those that better agreed with independent measurements related to RNA stability, such as the tAI, and translation efficiencies, such as the ribosome residence time measured by ribosome profiling. Based on these observations, we selected the most consistent mRNA half-life data-set and used the CSC to derive average values for individual genes (CSCg).

With this metric, we investigated the distribution of CSCg values across the yeast genome and the relationship between CSCg and protein translation. Overall, the results agreed with previous observations that linked a high proportion of optimal codons to mRNAs of highly abundant proteins, but we noted that genes that were only transiently overexpressed in response to stress conditions had CSCg values similar to those of constitutive genes with high expression levels. Finally, we used CSCs to identify and locate mRNA regions enriched in non-optimal codons and to examine how these sequences affected the translation efficiency. Our genome-wide analysis confirmed some of the assumptions derived from works using reporter systems (14). We observed that non-optimal codons were unevenly distributed across the mRNA sequence and that the occurrence of non-optimal sequence stretches increased the ribosome residence time and decreased the mRNA half-life. However, in contrast to observations from reporter genes, we found that the position of a single stretch of extremely non-optimal codons did not affect mRNA stability.

## MATERIALS AND METHODS

### Data sources

Coding sequences and annotation of *S. cerevisiae* were obtained from the *Saccharomyces cerevisiae* genome database (SGD ((https://www.yeastgenome.org) and Ensemble Genomes (http://ensemblgenomes.org/). We excluded 746 dubious ORFs and mitochondrial genes as defined in the Saccharomyces Genome Database from our analysis. We gathered 9 datasets from published studies measuring mRNA half-lives in yeast, namely Young (15), Brown (16), Hughes (17), Coller (11), Struhl (18), Weis (19), Gresham (20), Cramer (21) and Becskei (12). For most analyses, the Coller dataset utilized was the total RNA isolation dataset (11). Chemicals, such as phenanthroline and thiolutin, have been used to inhibit translation and consequently to address mRNA decay rates (22). Nevertheless, these chemicals have pleiotropic effects and inhibit a large number of enzymes. Therefore, we excluded all mRNA turnover measurements based on the use of chemical compounds to inhibit the RNA polymerase from our datasets.

**Table:** 

Data	Source	Identifier
tAI	dos Reis *et al.*, 2004	
Ribosome density at A site	Fang *et al.*, 2018;	
Ribosome density at A site	Gardin *et al.*, 2014	
Ribosome density at A site	Weinberg *et al.*, 2016	
tRNA abundance	Tuller *et al.*, 2010	
Frequency of optimal codons	CodonW-Saccharomyces Genome Database	
Protein abundance	Ho *et al.*, 2018	
mRNA abundance	Yassour *et al.*, 2009	
Reads per kilobase per million	Heyer and Moore, 2016	
Protein abundance	Kulak *et al.*, 2014	
Induced stress proteins	Breker *et al.*, 2013	
*S. cerevisiae* CHX-free Riboseq data	Gerashchenko and Gladyshev, 2014	SRR1520311
*S. cerevisiae* + CHX Riboseq data	Gerashchenko and Gladyshev, 2014	SRR1520315
*S. cerevisiae* CHX-free Riboseq data	Weinberg *et al.*, 2016	SRR1049521
*S. cerevisiae* CHX-free Riboseq data	Pop *et al.*, 2014	SRR1688545
**Software and algorithms**		
Codon stabilization coefficient gene (CSCg)	This paper	https://github.com/RodolfoCarneiro/CodonOptmality/tree/master/Sequence_mean_values
Shuffled genome	This paper	https://github.com/RodolfoCarneiro/CodonOptmality/tree/master/Shuffle
Localization of non-optimal/optimal codon stretches	This paper	https://github.com/RodolfoCarneiro/CodonOptmality/tree/master/Distribution

### Statistical analyses, correlations, and raw data

**Figure 1.**
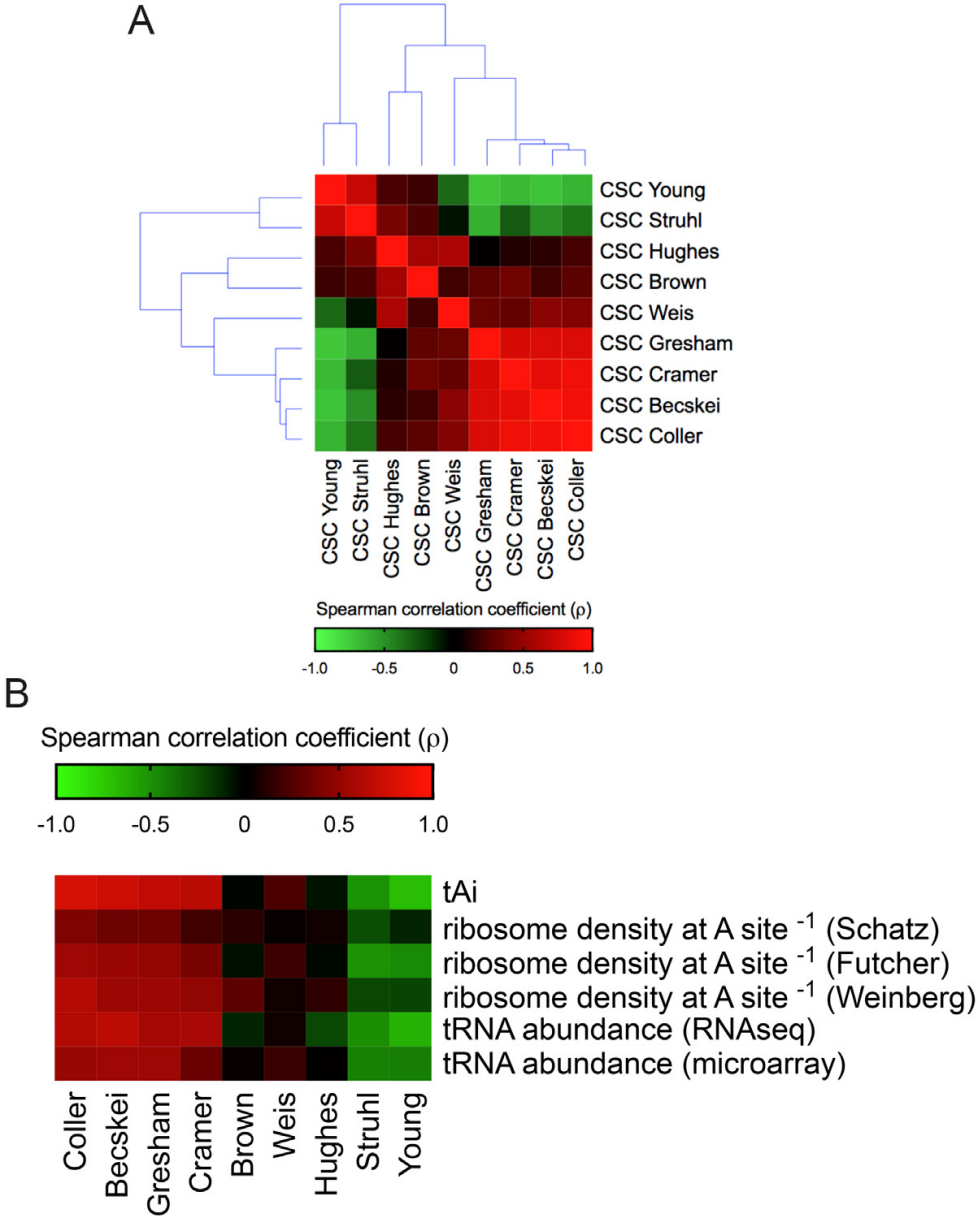
Comparison between different *S. cerevisiae* mRNA half-life datasets using the codon stabilization coefficient (CSC). (**A**) The CSC for each of the 61 amino acid coding codons equals the correlation coefficient (*r*) derived from Pearson's correlation between the frequency of occurrence of each codon in the transcriptome and the mRNA half-life values. Each mRNA half-life dataset generated 61 CSC values that were used to compare the studies. The heat map shows Spearman's correlation coefficients (ρ) ranging from –0.64 (negative correlation, green panels) to 1.00 (positive correlation, red panels). (**B**) Spearman's correlation between the CSCs derived from nine datasets with different cellular parameters: tAI (9), the inverse of the ribosome density at the A-site (27,32,33) and the tRNA abundance (27,28). The heat map shows Spearman's correlation coefficient (ρ) values ranging from –0.72 (negative correlation, green panels) to 0.70 (positive correlation, red panels). The raw data, sample size, *P* values, uncorrected critical ρ and Spearman's correlation coefficient (ρ) are presented in Supplementary Table S1.

**Figure 2.**
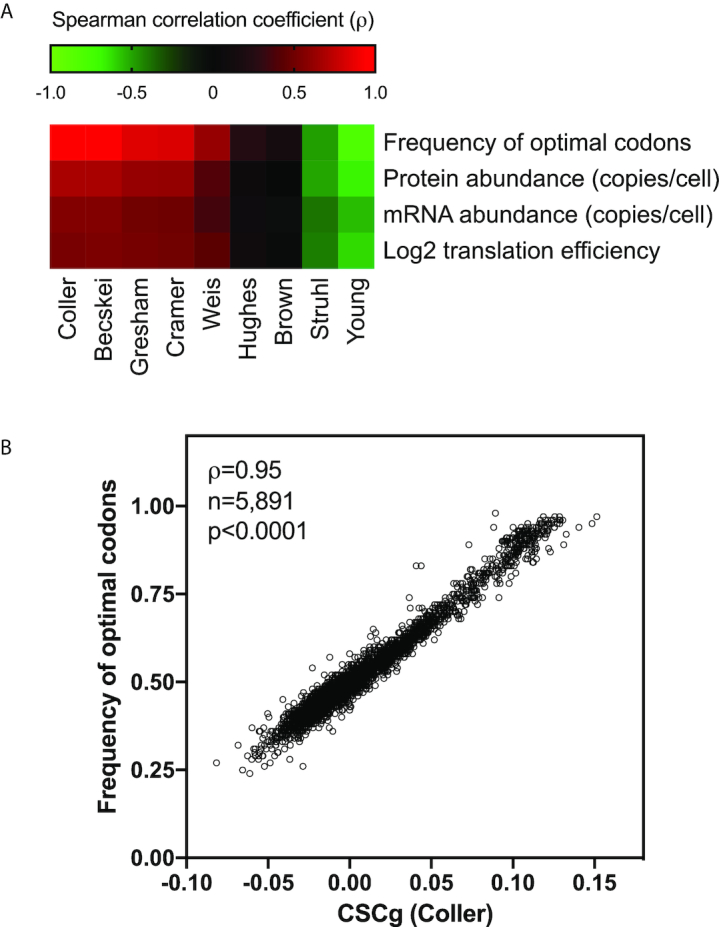
*Saccharomyces cerevisiae* individual gene CSCg calculation and its relationship with different parameters involved with gene expression. The sum of the CSCs of each codon in an mRNA divided by the length of the gene equals the CSCg, which is a metric that can be used to estimate the optimization of codon usage in a given sequence. We calculated the CSCg for 5891 yeast genes. (**A**) Spearman's correlation analysis between CSCgs calculated from the nine mRNA half-life datasets and other gene-specific parameters, such as the frequency of optimal codons (CodonW SGD), protein abundance (35), mRNA abundance (36) and translation efficiency (27). The heat map shows correlation coefficient (ρ) values ranging from –0.79 (negative correlation, green panels) to 0.95 (positive correlation, red panels). The raw data, sample size, *P* values, uncorrected critical ρ and Spearman's correlation coefficient (ρ) are presented in Supplementary Table S2. (**B**) As an example, we show the correlation between the CSCgs derived from Coller's dataset and the frequency of optimal codons according to John Peden's CodonW script available at SGD.

**Figure 3.**
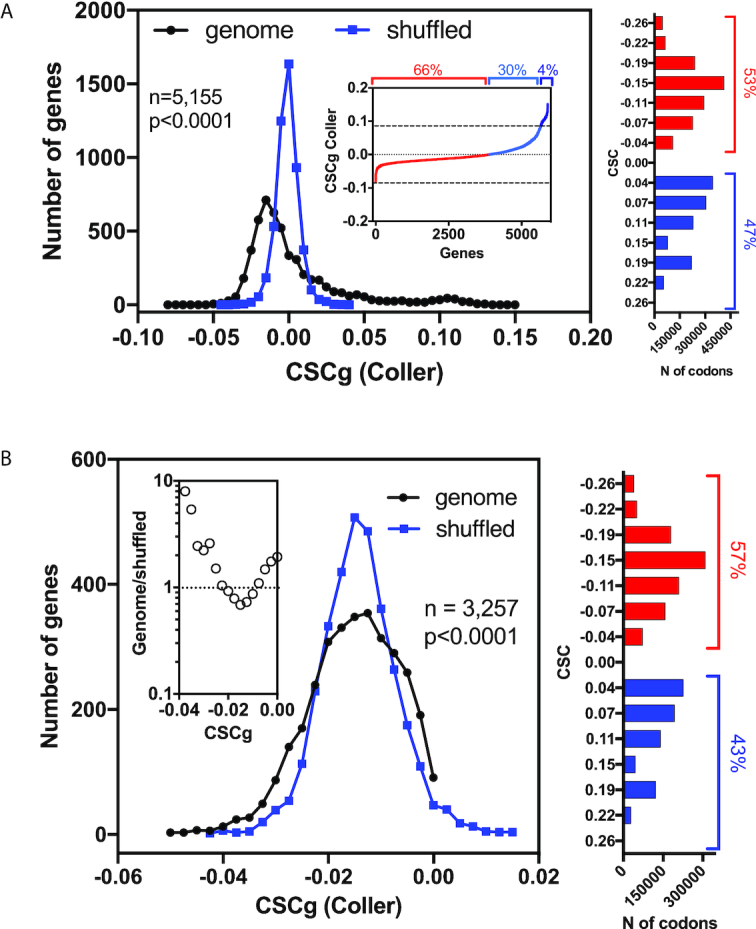
Distribution of *S. cerevisiae* CSCg values compared with a shuffled version of the genome. (**A**) The frequency distribution of CSCgs calculated from Coller's CSC is shown in black. The upper panel shows a cumulative distribution of this dataset. A shuffled version of the genome was generated by maintaining the same codon bias and protein sequence but randomizing the codon usage in each mRNA. The resulting CSCg distribution of the shuffled dataset is shown in blue. The frequency of the distribution of individual CSC values is shown on the right; this distribution was maintained in the shuffled version of the genome. (**B**) The subset of genes with CSCg values below zero (black lines) was compared to another shuffled mRNA dataset (blue line). This new shuffled dataset was generated as described in A, but this time the frequency of codons derived from the mRNA population with negative CSCg values (shown in the right panel) was used in the calculation. A ratio between the actual genome and the shuffled distribution is shown in the inset; note that the frequency of genes with CSCg values lower than 0 is ten times higher in the *S. cerevisiae* genome than in the shuffled genome. The *P*-values were calculated with the Kolmogorov–Smirnov test for both panels. The raw data, sample size, *P* values, and statistical analyses are presented in Supplementary Table S3.

**Figure 4.**
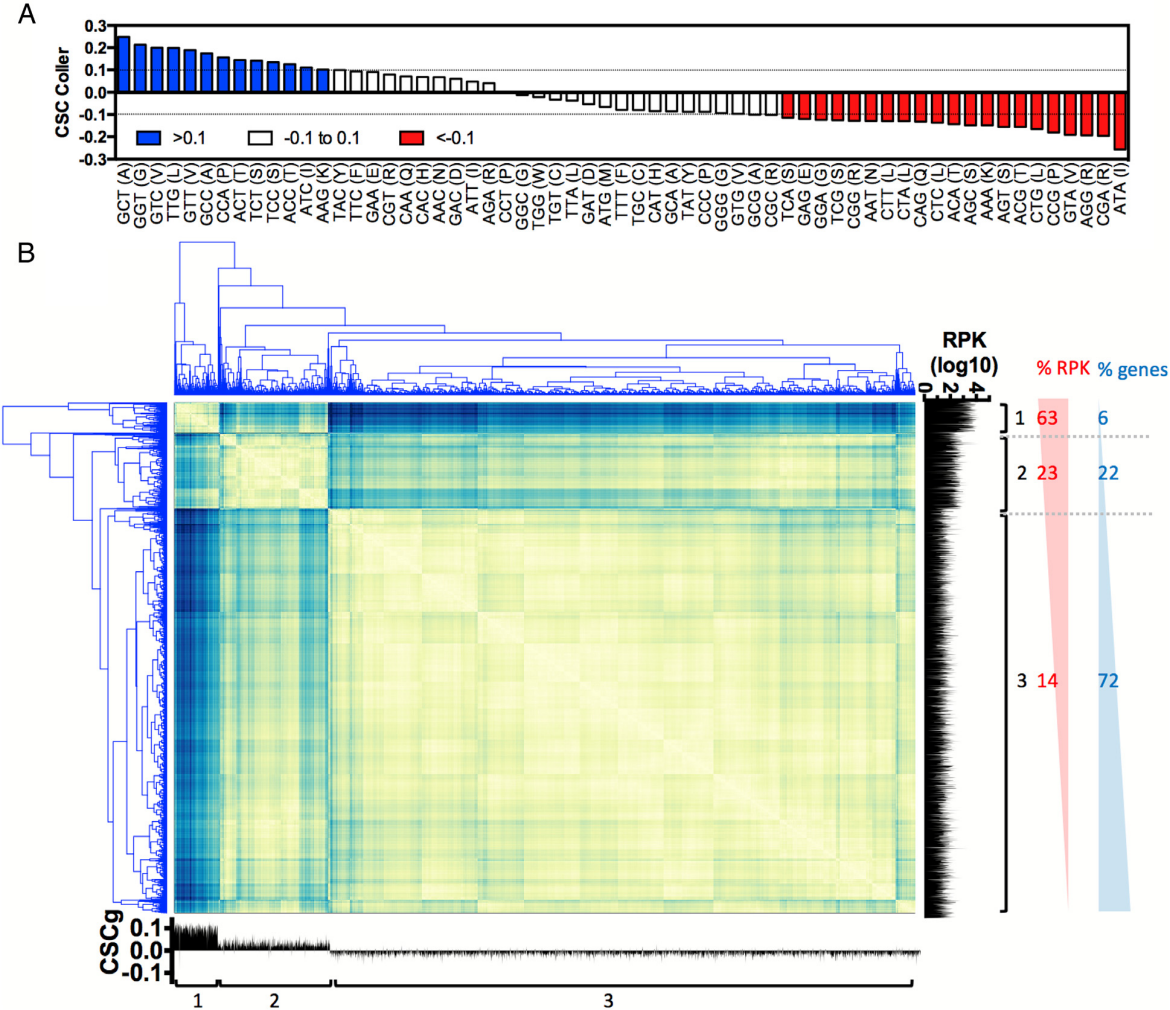
Evaluation of the mRNA codon composition and gene expression using the CSCg and CSC values. (**A**) The 61 codons were divided into three groups according to their CSC values: optimal codons are shown in blue (CSC > 0.1), neutral codons are shown in white (0.1 > CSC > –0.1) and non-optimal codons are shown in red (CSC < –0.1). (**B**) Distance matrix created from gene level clustering based on the proportion of optimal, neutral and non-optimal codons. Three main clusters were found. The number of reads associated with the ribosomes is shown on the right (RPK), and the CSCg for each gene is shown on the bottom. The raw data and the gene ontology analyses for panel B are presented in Supplementary Table S4.

**Figure 5.**
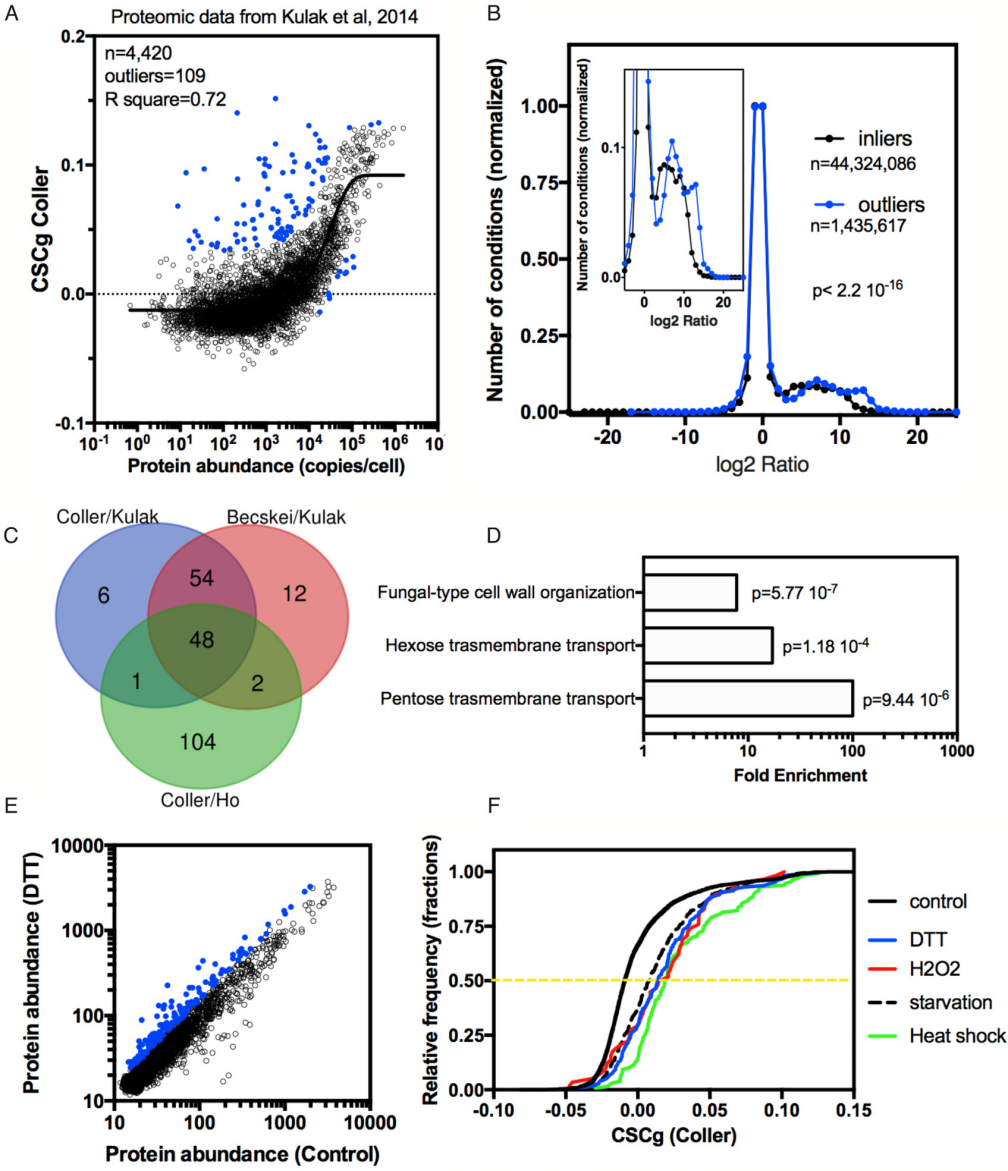
Correlation between CSCg and the protein copy number in *S. cerevisiae*. (**A**) The best-fit curve was used to calculate the correlation among the CSCg values (Coller's dataset) and the protein abundance (Kulak's dataset) (40). Outliers are highlighted in blue. (**B**) Plot showing the distribution of genes according to their expression regulation under 400 different experimental conditions (data obtained from the SGD). The inlier genes are shown with a black line, and outlier subsets of genes are shown with blue lines. The inset shows the overexpressed population on a different scale. Note that the outliers presented higher induction levels and were overexpressed in more conditions than the inlier group. The *P*-value was calculated with the Kolmogorov–Smirnov test (Supplementary Table S5B). (**C**) The same analysis described in panel A was performed to compare other datasets (i.e. CSCg values from Coller's dataset vs protein abundance from Ho's dataset (41) (Supplementary Figure S6) and CSCg values from Becskey's dataset versus protein abundance from Kulak's dataset (Supplementary Figure S5)). The Venn diagram of outliers identified in these three analyses showed 48 genes in common. (**D**) Gene ontology of the 48 genes identified in panel C. For the subsequent experiments, we analyzed a different subset of proteins that were upregulated under different stress conditions. (**E**) As an example, we show the correlation plot used to select the subset of proteins induced by DTT (43). Proteins with an induction level of more than 1.5-fold were selected (blue dots). (**F**) Cumulative distribution of the CSCg values of genes coding the proteins induced more than 1.5-fold by different stresses (43) was compared to that of all genes (control). The p-value calculated with the Kolmogorov–Smirnov test was <0.0001 for each stress condition analyzed versus the control (see statistical details in Supplementary Table S5B).

**Figure 6.**
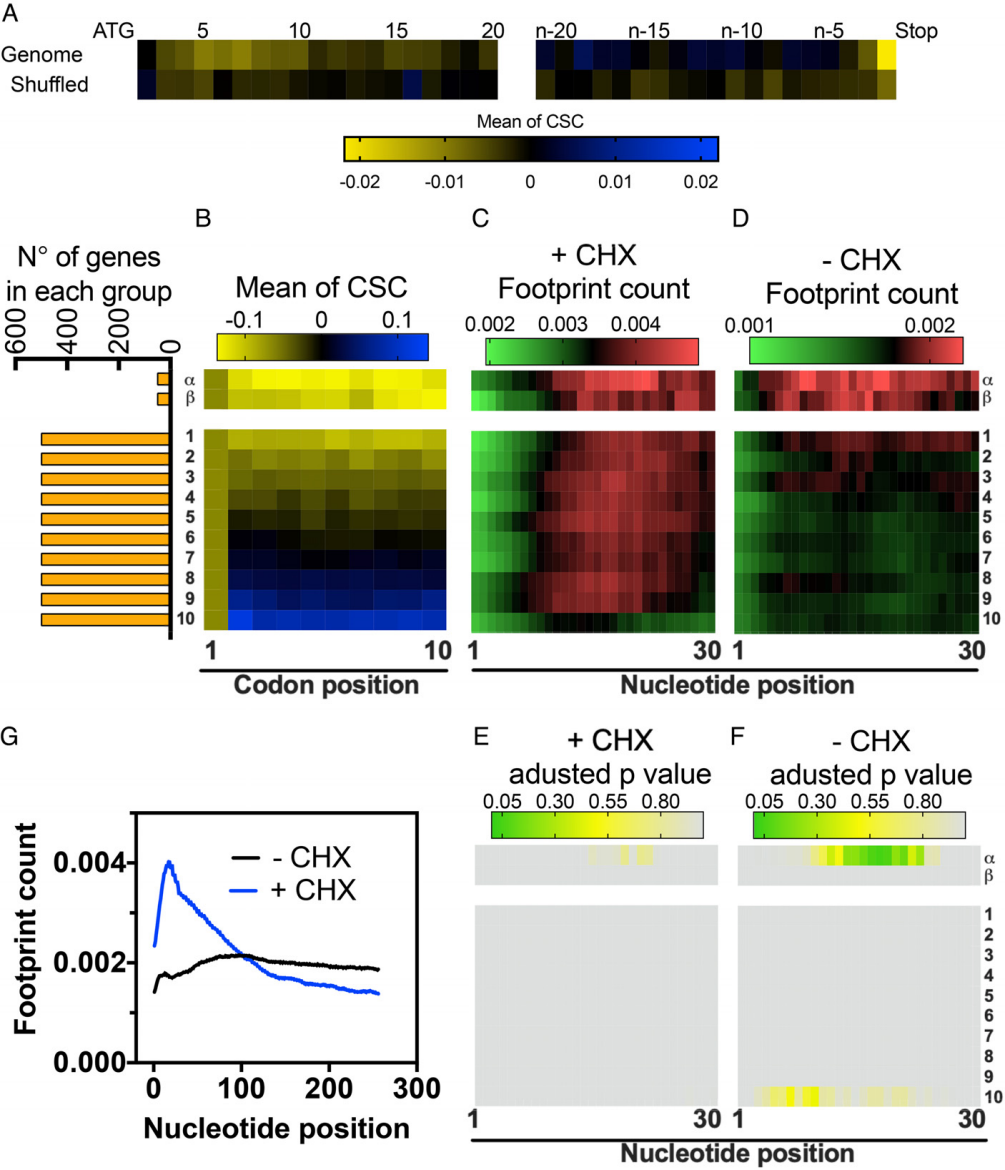
The enrichment of non-optimal codons near the translation initiation site leads to an increase in ribosome density in a small subset of genes. (**A**) Average CSC value according to each codon position in the yeast mRNAs. The tile strip on the left represents the first 20 codons, whereas the strip on the right represents the last 20 codons. The heat map shows the mean CSC values ranging from 0.02 to -0.02. A shuffled version of the yeast genome was analyzed as a control. (**B**) The full yeast genome was subdivided into 10 groups with 500 genes each, organized according to the average CSC of the first 10 codons. The first group (1) contains genes with the lowest average CSC while the last group (10) contains genes with the highest average CSC. The α and β groups are composed of the first 50 sequences with the lowest CSC and the next 50 lowest CSC, respectively. The orange bars show the number of genes in each group. The average ribosome footprint count of each group was derived from experiments performed in the presence (+CHX) (**C**) or in the absence of cycloheximide (–CHX) (**D**). For each gene, the number of reads per base was normalized to the total number of reads in a 500-nucleotide window after the ATG of the same gene. The variations in footprint count for each of the groups shown in panels C and D were analyzed by multiple *t*-tests using the Holm–Sidak method; the adjusted *P*-values are shown using a heat map in panels (**E**) and (**F**), respectively. Panel (**G**) shows the normalized average ribosome footprint counts for all yeast genes in the presence or absence of CHX.

**Figure 7.**
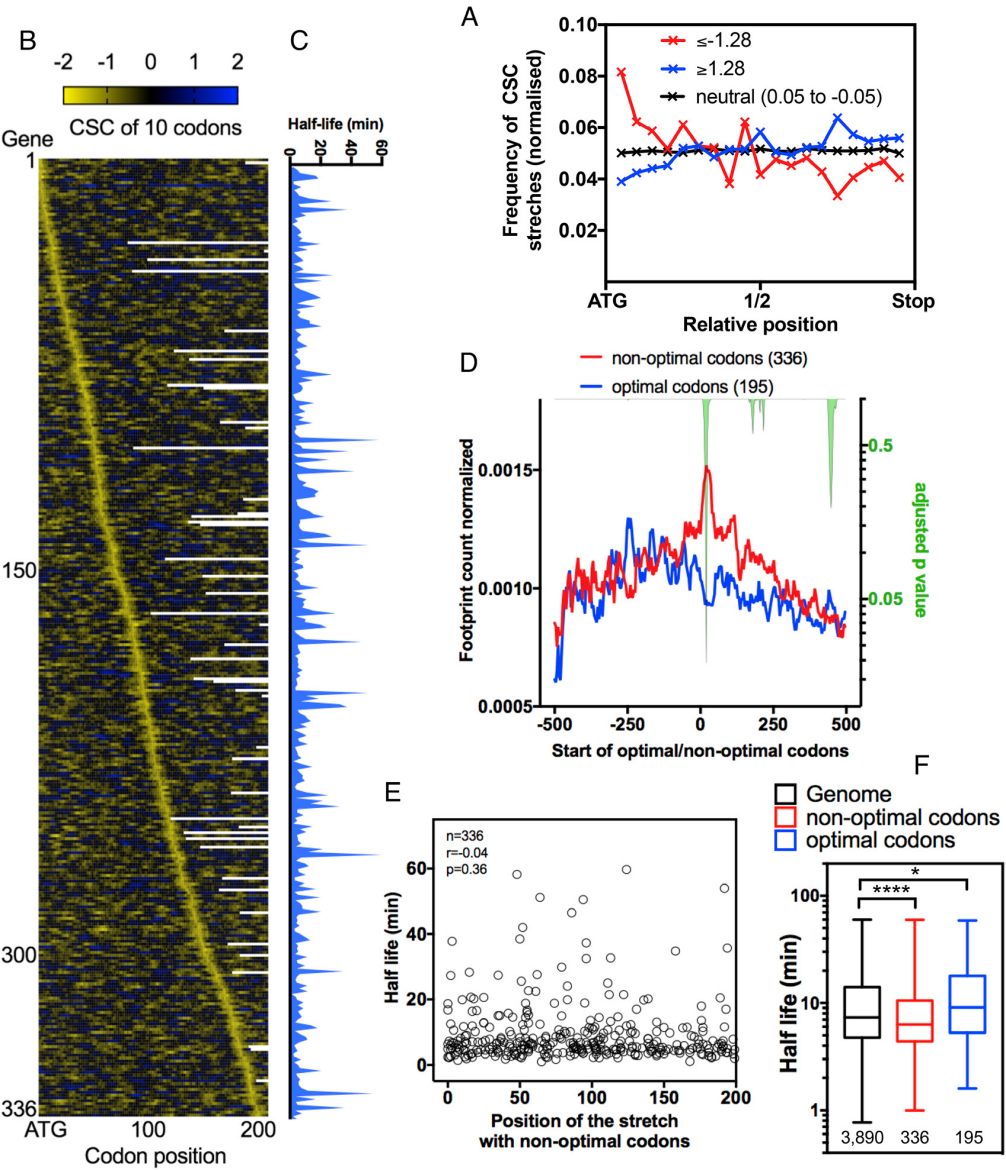
Occurrence and position of non-optimal/optimal codon stretches in yeast mRNAs and their impact on translation. (**A**) The frequency of stretches of 10 consecutive codons with extremely low CSC values (≤–1.28) (red line) across the mRNA sequence. Neutral stretches (0.05 ≥ CSC ≥ –0.05, black line) and optimal stretches of CSCs (≥1.28) (blue line) were used as the control groups. Dunn's multiple test comparisons; **** ≤–1.28 versus neutral (0.05 to –0.05), **** ≤–1.28 versus ≥1.28 and **** ≥1.28 versus neutral (0.05 to –0.05). See details of the statistical analysis in Supplementary Table S7A. (**B**) mRNA sequences containing a 10-codon stretch with CSC values ≤–1.28 were aligned according to the position of the non-optimal stretch from the start codon. The heatmap depicts the sum of the CSC values in a ten-codon window. Panel (**C**) shows the mRNA half-lives (11) of the genes presented in panel 7B. (**D**) The average ribosome footprint density of the genes presented in panel 7C from the stretch of non-optimal codons (position 0). The number of reads at each position was normalized to the total number of reads in a 500-nucleotide window before and after the stretch. Genes with optimal stretches of CSC (≥1.28) (blue line) were used as the control group. The right y-axis represents the adjusted p-value (green area) calculated for each nucleotide position by multiple *t*-tests using the Holm-Sidak method. (**E**) The mRNA half-life values (11) were plotted according to the position of the non-optimal codon stretch (CSC ≤ –1.28) from the ATG. No correlation was found between the non-optimal codon position and the mRNA half-life. (**F**) The half-life (11) of the gene categories used in panels A and D. Kruskal–Wallis test, ****<0.0001, *0.017.

The raw data used to create Figures 1–7 and for the statistical analyses, including sample size, *P* value, *r* value, ρ value, uncorrected critical *r*, uncorrected critical ρ and D’Agostino & Pearson normality test calculations, when used, are presented in the Supplementary Tables S1–S7. For Figures 3A, B, 5B and F, the Kolmogorov–Smirnov test was used (Supplementary Tables S3A, S3B, S5B and S5F). For Figure 5A, the best fit equation found was a Sigmoidal, 4PL, *X* is log(concentration) equation; the outliers were identified with *Q* = 1% with a confidence level of 95%. All statistical analyses were performed with GraphPad Prism 7 except for the Kolmogorov-Smirnov test used for Figure 5B, which was performed with the R software.

### Gene ontology

The gene ontology analyses were performed in the Gene Ontology Consortium (http://geneontology.org/).

### Codon stabilization coefficient (CSC) calculation

We calculated the codon stabilization coefficient (CSC) for each codon based on the method proposed by Coller *et al.* (11). The CSC value of a given codon is represented by the Pearson correlation coefficient (*r*) between the mRNA half-life of a gene and the proportion of the given codon in that gene, which is calculated by

(1) \begin{equation*}{N_{ij}} = \frac{{{C_{ij}}}}{{{l_i}}}{\rm{\ }}\end{equation*}

where *N_ij_* is the proportion of codon *j* in gene *i* normalized by the size of the gene measured in codons, *C*_*ij*_ is the absolute number of copies of codon *j* in gene *I*, and ℓ_*i*_ is the length of gene *i* reported in the number of codons.

To calculate the proportion of a codon in a given gene, we implemented an algorithm that analyzed an entire dataset of the yeast genome downloaded from the SGD. The algorithm uses as input a list with every gene in a genome containing a header (with the gene identification code) and the sequence of the nitrogenous bases of each gene. Then, the algorithm compares each group of three bases representing the frame of the ORF to a library containing all 61 codons and adds 1 to the count of that codon if it is a match. At the end of each gene, the proportion of each codon is calculated using Equation 1. This process is repeated for every gene. As output, our algorithm creates a comma-separated values file (.csv) with the calculated proportions of codons for each gene from the input file.

### Mean codon stabilization coefficient (CSCg) calculation

To study the effect of codon usage in yeast genes, we designed and implemented another algorithm that calculated the mean value of CSC for each gene (CSCg) with the following equations:

(2) \begin{equation*}{\rm CSC}{g_i} = \mathop \sum \limits_j \left( {{{\rm CSC}_j} \cdot \frac{{{C_{ij}}}}{{{l_i}}}} \right)\ \end{equation*}

(3) \begin{equation*}{\rm CSC}{g_i} = \mathop \sum \limits_j \left( {{{\rm CSC}_j} \cdot {N_{ij}}} \right)\end{equation*}

where CSC*g*_*i*_ is the mean CSC for gene *I*, CSC_*j*_ is the CSC value for codon *j* and *N*_*ij*_ is the proportion of codon *j* in gene *i* normalized to the size of the gene measured in codons (Equation 1).

### Shuffled genome

Data from the original genome were compared to that from a shuffled genome to evaluate whether the codon usage of the genes was random or evolutionarily selected. For that purpose, we designed and implemented a new algorithm that exchanged codons with their synonymous codons. The algorithm works in two steps.

First, it counts the total number of each codon in all target ORFs and arranges them according to the amino acid they translate. Then, it rereads every target ORF and exchanges each codon for a codon that translates to the same amino acid. The shuffled codon is selected randomly and proportionally to the total number of copies of that codon (counted in the first step). The random genome generated by this algorithm preserves the number of copies of each codon as well as the amino acid sequence of the original genome.

### Localization of non-optimal/optimal codon stretches

We designed and implemented an algorithm that summed the CSC (CSC Coller) in a determined number of consecutive codons in an mRNA sequence. In this work, we were interested in retrieving sequences that contained 10-codon stretches with summed values ≤–1.28, which is a parameter that has been previously associated with mRNA destabilization (14). We used genes that contained 10-codon stretches with summed values ≥1.28 as a control. The program returns a list with the mRNAs containing the non-optimal or optimal stretches and the position of non-optimal or optimal codons relative to the ORF length. Stretches with 10 non-optimal or optimal codons were identified (6386 in 1404 genes for optimal stretches and 1702 in 1208 genes for non-optimal stretches). Then, genes with more the one stretch were removed. Finally, the genes with stretches before codon 200 and the mRNA half-life determined were analyzed by ribosome profiling (195 genes with optimal stretches and 336 genes with non-optimal stretches). We compared the ribosome profiles normalized for genes with non-optimal stretches versus optimal stretches. Statistical analyses were performed with multiple *t*-tests using the Holm–Sidak method, and the adjusted p-value was calculated for each point (GraphPad Prism 7).

### Ribosome profiling data

*Saccharomyces cerevisiae* ribosome profiling data were treated as described previously (23,24). The data were analyzed as described by Ingolia and collaborators (25) except that the program used here was Geneious R11 (Biomatter Ltd., New Zealand) instead of the CASAVA 1.8 pipeline. The data were downloaded from GEO, and the adaptor sequence (CTGTAGGCACCATCAAT) was trimmed. The trimmed FASTA sequences were aligned to *S. cerevisiae* ribosomal and noncoding RNA sequences to remove rRNA reads. The unaligned reads were aligned to the *S. cerevisiae* S288C genome deposited in the Saccharomyces Genome Database. First, we removed any reads that mapped to multiple locations. Then, the reads were aligned to the *S. cerevisiae* coding sequence database allowing two mismatches per read. We normalized the coverage within the same transcript by dividing each nucleotide read by the sum of the number of reads for each gene in a given window. A window of 500 nucleotides before and after the beginning of a non-optimal/optimal stretch was used to calculate the number of reads of each gene. This step is important because longer genes possess lower normalized numbers of reads than shorter genes if the total number of reads is used to normalize.

### Clustering analysis

All clustering analyses were performed by the Euclidean distance using the Orange 3 software. For Figure 4B, a cluster analysis at the gene level was performed based on a proportion of optimal, neutral and non-optimal codons, and the distance among genes was calculated by the Euclidean metric. The distance measures were visualized in a clustered distance matrix (Orange 3).

## RESULTS AND DISCUSSION

### CSC calculation showed the most correlated *S. cerevisiae* mRNA half-life datasets

In *S. cerevisiae*, mRNA half-life experiments usually involve a time-resolved analysis of the mRNA levels after a procedure to block transcription or label the mRNA *in vivo* (Table 1). In the transcriptional inhibition method, RNA polymerase II is inactivated, usually by heat shock, to interrupt the synthesis of new mRNAs. In the gene activation control approach, the promoter of each gene is substituted by a promoter that can be inhibited by the addition of a substance in the medium (i.e. GAL or the TET promoter). For *in vivo* metabolic labeling, modified nucleotides are introduced into the cell medium and incorporated by the cells into the RNA. The modified mRNA can be recovered by immunoprecipitation or pulled down by streptavidin beads. We selected nine global quantitative studies aimed at determining yeast mRNA half-lives, 5 of which used the transcriptional inhibition method, three *in vivo* metabolic labeling and one the gene control method (Table 1). In agreement with previous work (12,13), we observed that the ranges of mRNA half-life values varied depending on the dataset (Supplementary Figure S1A), yielding generally poor Spearman's correlation coefficients (ρ values ranging from –0.25 to 0.75) (Supplementary Figure S1B). Moreover, we found little to no correlation between these mRNA half-life datasets and independent parameters, such as protein expression, translation efficiency, and mRNA abundance (data not shown). This scenario made selecting a particular dataset for further analysis difficult.

**Table 1.** Description of the mRNA decay experiments analyzed in this work

Name	Ref	Method	Quantification	Yeast strain medium	mRNA enrichment	cDNA synthesis
Young	Holstege *et al.*	rbp1–1/TI	Microarray	Z460 YPD	Oligo dT-beads	
Brown	Wang *et al.*	rbp1–1/TI	Microarray	Y262 YPD		Random primers^a^ or Oligo dT
Hughes	Grigull *et al.*	rbp1–1/TI	Microarray	YF2475 YPD	Oligo dT-beads	Oligo dT
Coller	Presnyak *et al.*	rbp1–1/TI	RNA-seq	yJC244 SD	Bead-based rRNA depletion or Oligo dT-beads	Random primers
Struhl	Geisberg *et al.*	rbp1-frb/TI	DRS	JGY2000 YPD	Silica-membrane for total RNA	
Weis	Munchel *et al.*	4tU chase	RNA-seq	W303–1A SD	Oligo dT-beads	Random primers
Gresham	Neymotin *et al.*	4tU chase	RNA-seq	FY4 SD	Bead-based rRNA depletion	Random primers
Cramer	Sun *et al.*	4tU labeling	Microarray	GRY3020 YPD	Glass-fiber filter purification rRNA depletion	Oligo dT
Becskei	Baudrimont *et al.*	Gene control	qPCR	BY4741 SD	Silica-membrane for total RNA	Gene-specific primers

^a^The dataset used herein was obtained with random primers.

The codon stabilization coefficient (CSC) is the correlation coefficient between the frequency of occurrence of each codon in mRNA and the mRNA half-life (11). An example of the CSC calculation for the codon GCT is shown in Supplementary Figure S2. Coller and colleagues observed that CSC values had a significant positive correlation with the tRNA Adaptive Index (tAI), which is a metric that ranks the codons according to the efficiency of tRNA usage by the ribosome and is widely used to calculate codon adaptability (9). The close association between the RNA half-life and codon optimality was also observed by independent means in both yeast (11) and bacteria (26).

We used nine different mRNA half-life datasets to calculate the CSC of each of the 61 codons using the same method as Coller *et al.* (Figure 1A); then, we calculated how the different CSCs correlated between these different studies (Figure 1A and Supplementary Figure S3). We observed that four studies that used different methodologies to quantify mRNA half-lives clustered together (i.e. Coller, Becskei, Gresham and Cramer). The correlation coefficients between the CSCs calculated from these four studies ranged from 0.78 to 0.91 (Figure 1A).

Next, we compared the CSC score of each codon calculated from published datasets with independent metrics used to estimate codon efficiency/adaptability and translation efficiency. In addition to the aforementioned tAI (9), we used previously published tRNA abundance quantifications based on RNA-seq (27) or microarray (28) as well as data derived from ribosome footprint profiling. This methodology was created by Jonathan Weissman in 2009 and was based on the deep sequencing of ribosome-protected mRNA fragments (29). During translation, each ribosome encloses an exactly 28-nt portion of the mRNA to protect it against RNAase digestion. This enclosure allows the protected fragments to be sequenced. This approach generates a map at nucleotide resolution of translational activity for transcribed genes (25). Importantly, all ribosome profiling data used herein were obtained in the absence of a translational inhibitor in the cell media, since this treatment distorts the ribosome profile because initiation continues even though elongation is blocked (23,24,27,30,31). Coller, Becskei, Gresham and Cramer's CSC values showed good positive correlations with the tAI, with Spearman correlation coefficients (ρ) ranging from 0.65 to 0.77, whereas the CSCs from other studies had low or even negative (ρ) values (–0.69 to 0.23) (Figure 1B). The same pattern was observed for correlations with different tRNA abundance measurements. We took advantage of the nucleotide resolution of ribosome profiling to examine whether the difference in occupancy at the A-site for each codon correlated with the CSC calculated for each dataset. In principle, if a codon is optimal, then the ribosome occupancy of this codon must be shorter than that of a non-optimal codon; therefore, we used the inverse of the ribosome residence time to correlate with the CSC (Figure 1B). We observed that the CSCs that most agreed with three independent experiments measuring the codon occupancy time by ribosome profiling (27,32,33) were again the Coller, Becskei, Gresham, and Cramer datasets (Spearman correlation coefficients (ρ) ranging from 0.19 to 0.62, Figure 1B). Very recently, the ribosomal A-site decoding rate was shown to impact normal mRNA decay in yeast, thereby reinforcing the correlation between CSC and codon occupancy (34).

When the mRNA half-life values were directly compared, the correlation was strongly impacted by discrepancies caused by different methodologies, outliers, population sizes, strain choices and other factors (Table 1). The use of the CSC diluted the contribution of individual mRNA half-life values to the correlation analysis and thus helped to identify the more congruent studies. A close inspection of the experimental conditions of the nine studies used in our analysis suggested that methods that avoided an enrichment step using oligo dT-beads tended to have better CSC correlation values, even when the translation inhibition and/or mRNA quantification methodologies were different. In fact, methods based on poly-A mRNA capture are known to possess some limitations, such as down sampling deadenylated mRNA and mRNAs containing certain stable secondary structure elements (11). Moreover, by varying only the mRNA enrichment methodology (ribosomal RNA depletion × poly-A fraction), Coller *et al.* observed different half-life values for 92% of the transcripts. The Spearman correlation coefficients between the CSCs calculated using the poly-A fraction from Coller's dataset and the tAI, ribosome density at the A site, and tRNA abundance were as poor as the values obtained with Young's dataset (ρ values ranging from –0.32 to 0.04 and from –0.69 to –0.11, respectively) (data not shown). Although the CSC calculation demonstrated common patterns among different mRNA half-life datasets, this metric cannot be used as a validation method for a given mRNA decay dataset.

With the CSC for every codon, we generated a mean CSC value for a given mRNA sequence termed the CSCg. This metric can be used to rank all yeast genes according to their stability. The CSCg values derived from the nine datasets were compared with the frequency of optimal codons (CodonW- SGD), protein abundance (35), mRNA abundance (36), and transcriptional elongation (27) (Figure 2A). The striking correlation between the frequency of optimal codons and the CSCg values calculated using Coller's data set is shown in Figure 2B. While CSCg is derived from mRNA stability data, CodonW is defined using a multivariate model of various codon supply-and-demand scores such as CAI, tAI. These scores are sought to affect ribosome dwell time and translation efficiency. The fact that Coller's CSCg and CodonW generate equivalent scores for yeast genes (Supplementary Table S2) strengthens the assumption that a metric derived from mRNA half-lives is a good indicator of codon optimization and reinforces the connection between codon choice, translation efficiency and mRNA half-life. Therefore, CSCg can be used to estimate mRNA stability and translation efficiency.

Coller's mRNA half-life measurement generated CSC and CSCg values that better correlated with independent experimental measurements linked to mRNA stability, translation and codon optimality (Supplementary Table S2); moreover, Coller's CSC was in good agreement with the studies of Becskei, Gresham, and Cramer. Therefore, we focused on these datasets in the next steps of this work. However, a good correlation between two CSCs derived from different studies can mask essential differences in the individual mRNA half-life values obtained by each research group. The discrepancy in absolute values between two studies with highly correlated CSCs is probably an inevitable consequence of using different experimental methodologies to determine mRNA half-lives.

### The occurrence of genes with extremely high or low CSCg values in the yeast genome is not random

We used the CSCg (Coller's dataset) to characterize the distribution of yeast mRNAs according to their stability. We observed that the majority of genes presented slightly negative CSCg values (non-optimal CSCgs) (Figure 3A, black circles). The cumulative distribution shown in the inset revealed that two-thirds of the CSCgs (66%) were non-optimal (Figure 3A, inset, red line), whereas one-third (34%) were optimal (Figure 3A, inset, blue line), with ∼4% of the genes presenting a high level of optimality (>0.1) (Figure 3A, inset, dark blue line). This pattern is reminiscent of other studies that used alternative metrics to rank yeast genes according to their codon optimality. This result was expected, considering the high correlation between the CSC and CSCg values and other metrics, such as the tAI and the frequency of the optimal codons (Figure 1B and Figure 2A). Nevertheless, we extend these analyses by testing whether the characteristic non-Gaussian distribution with long tails seen in Figure 3A results from the regulatory role exerted by the codon composition on the final biological function of each transcript. For this purpose, we created a shuffled version of each yeast mRNA sequence. We maintained the proportions of all 61 amino acid codons (genomic codon bias) as well as the final protein sequence but randomized the codon choice for each transcript. The CSCg values of the mRNAs with random codon choices (but the same overall codon bias) generated a more uniform distribution than that of the original mRNA population (Figure 3A, blue squares).

The dramatic difference between the real and artificial mRNA CSCg distributions underscores the impact of codon usage on mRNA biology. Abundant proteins tend to be coded by mRNAs that are enriched in optimal codons (37,38); therefore, we expected to find a long tail with highly positive CSCg values (Figure 3A). However, we did not expect to see a population of mRNAs enriched in non-optimal codons (negative values below -0.05; Figure 3A genomes × shuffled). Therefore, we decided to evaluate whether this subset of sequences with very low CSCg values was merely a consequence of having a group of transcripts that concentrated the codons with high CSC values. For this purpose, we selected genes with CSCg values lower than 0 (3257 genes), calculated the frequency of each codon in this population, and shuffled the mRNA codons as described in Figure 3A. However, for this analysis, we used the codon bias from the mRNAs with CSCg values lower than 0 (right panel Figure 3B). With this adjustment, we simulated a possible influence of the depletion of highly optimal codons on the distribution of the CSCg values on this population. We observed that the actual genome still contained more sequences with extremely low CSCg values than the shuffled version of the mRNA (Figure 3B, compare the negative values of the shuffled × genome). With this result, we concluded that codon usage probably played a regulatory role in sequences containing both optimal and non-optimal codons.

### Highly abundant constitutive proteins have mRNAs with the highest CSCg values, but genes that are only transiently upregulated in response to stress also present high CSCgs

The low expression of heterologous proteins coded by mRNA sequences with low codon usage provided robust evidence of the impact of codon adaptation on protein translation (4). Moreover, recent studies in yeasts suggested that codon optimality played an essential role in regulating protein expression, especially for mRNAs coding proteins with high expression levels (4). However, how far mRNA codon composition and stability can tune the endogenous protein translation efficiency is less clear.

To address how CSC relates to the protein translation efficiency, the 61 amino acid codons were arbitrarily classified into three categories based on their CSC values (Coller's dataset). We considered codons with a CSC higher than 0.1 optimal codons, whereas codons with a CSC lower than –0.1 were non-optimal codons (Figure 4A). Codons with CSCs in the range of –0.1 to 0.1 were considered neutral codons (Figure 4A). Then, we performed a gene-level cluster analysis of all yeast genes according to their CSC compositions (Figure 4B). A distance matrix was created, and three main groups were found. These clusters were analyzed regarding gene expression using previously published ribosome profiling data (Figure 4B, right plot) (39). Cluster 1 comprised genes mainly formed by optimal or neutral codons and <10% of the non-optimal codons. Although <6% of yeast genes were found in this category, they were responsible for approximately 63% of all transcripts being translated in the cell (Figure 4B). Genes in cluster 2 comprised up to 23% of non-optimal codons and represented 22% of genes and 23% of the translated sequences, respectively (Figure 4B). Finally, the third cluster grouped sequences with up to 50% non-optimal codons. The majority of yeast genes (72%) fell in this cluster, but they represented only 14% of all transcripts being translated in the cell (Figure 4B). A small number of genes are responsible for most of the transcripts and they are enriched with optimal codons, confirming the relationship between codon optimality, a long RNA half-life and higher translation levels. A similar profile was also observed when the CSC was derived from Becskey's dataset (Supplementary Figure S4). Gene ontology analysis of the three groups revealed that genes with optimal codons (cluster 1) were enriched in functional classes related to translation, rRNA binding and RNA binding, among others (Supplementary Table S4). The non-optimal genes (cluster 3) were enriched in genes with histone acetyltransferase activity and protein kinase activity, among others (Supplementary Table S4).

The results from Figure 4B agreed with previous data showing that constitutive highly expressed proteins have a high content of optimal codons. Indeed, when the CSCg values were correlated and plotted against the protein abundance estimated by proteomics (40), we observed a positive correlation (Figure 5A). All proteins with more than 10^5^ copies/cell had mRNAs with CSCg values higher than 0. Nevertheless, some low abundance proteins (<10^3^ copies/cell) were produced by mRNAs with high CSCg values (>0.1). In this section, we investigated the functional roles of this protein population. One possible explanation is that these genes are optimized for high expression but under a different condition than that used in Figure 5A. To test this possibility, we divided the yeast genes into two groups (inliers, black dots versus outliers, blue dots; *Q* = 1%, 95% confidence interval) and compared the fold change in gene induction/repression under >400 conditions using information from the SGD. We observed that the outlier group of genes tended to have higher levels of induction in a higher number of conditions than the inlier group (Figure 5B).

Because Coller's mRNA half-lives were evaluated after a mild heat shock, we repeated the CSCg to protein expression correlation analysis using Becskei's mRNA half-life data set, whose methodology did not involve temperature changes (Table 1). We also tested an independent proteome dataset (41). More than 80% of the outliers identified using Coller's and Becskei's CSCg to protein expression correlation analysis were identical (Supplementary Figure S5). The outlier population was also significantly more susceptible to upregulation than the inliers regardless of whether the CSCg or proteomic dataset was used (Supplementary Figures S5 and S6). A Venn diagram of the outliers identified using different CSCg and protein expression datasets revealed 48 genes in common (Figure 5C). A gene ontology enrichment analysis of these genes that were low abundance but had high CSCg values revealed that proteins involved in carbohydrate transporters and cell wall organization were overrepresented (Figure 5D). In yeast, the expression of some hexose transporters is strongly regulated by the presence of glucose; for some transporters, the induction can be as high as 300-fold (42).

We also calculated the CSCg values of mRNAs coding proteins that were previously shown to be upregulated above the threshold of 1.5-fold induction under different stress conditions. Figure 5E shows an example of the analysis used to identify proteins overexpressed in response to DTT (43). Cumulative distributions comparing CSCg values of genes that were found to be upregulated by DTT and other stress conditions with the overall yeast mRNA CSCg values are shown in Figure 5F. The same analysis was repeated with CSCgs generated by different mRNA half-life datasets obtained using diverse methodologies (Supplementary Figure S7). Regardless of which CSC dataset was used, we observed that the CSCg values of stress-induced genes were higher than those of the total proteome (Figure 5F and Supplementary Figure S7). These analyses indicated that for many stress inducible genes, high CSCg scores correlated with high protein and mRNA levels observed under stress conditions, no matter the CSCg was based on mRNA half-lives from cells growing in optimal conditions. Since mRNA-decay rates can drastically change in response to stress it would be interesting to experimentally measure the stability of stress responsive mRNAs and evaluate at which extent different growth conditions can affect CSC values.

### Non-optimal codons lead to pauses in translation, but the effect on the mRNA half-life is independent of its position in the mRNA sequence

Previous reports showed that non-optimal codons were concentrated in both the 3′ and 5′ regions in several genes (28,44). The biological explanation for this observation relies on the efficiency with which a ribosome translates these codons (28). The ribosome ramp hypothesis claims that highly expressed genes possess a subset of non-optimal codons in their 5’ mRNA regions, which are necessary to slow down translation and thus avoid ribosome jams and minimize the cost of protein translation. However, the ribosome profiling data used to support the ribosome ramp hypothesis were collected in the presence of cycloheximide prior to cell lysis. As mentioned before, this protocol introduces artifacts in the data, especially in the region near the ATG (Figure 6G, 27, 30, 31). Using the CSC as a metric, we measured the distribution of codons in the yeast genome. Similar to the tAI (28), we observed an enrichment of non-optimal codons from codon 3 until codon 10 (Figure 6A). A more discrete enrichment was also observed in the region close to the stop codon (Figure 6A). To verify whether enrichment of non-optimal codons near the initiation site are implicated in the ramp formation we calculated the average CSC value of the first 10 codons and separated the yeast genes in 10 groups of approximately 500 genes, based on their scores (Figure 6B, groups 1–10): group number one contains the genes with the lowest codon optimality while group number ten contains the genes with the highest CSC values in their first 10 codons (Figure 6B, groups 1–10). In order to analyse the effect of sequences with extremely low CSC sores, we separated the 100 genes with the lowest scores into two small groups: α group, with the first 50 genes, and β group, with the next 50 genes (Figure 6B, α and β groups). Next, we compared the average ribosome footprint count per nucleotide of each group in the presence or absence of cycloheximide (Figure 6C and D, respectively). Figure 6C shows that all groups had a steady increase in footprint counts from the start codon, what characterizes the ramp previously described by others (28). However, none of the groups was different from the average genome (Figure 6E). When the ribosome profiling was performed in the absence of cycloheximide, the increase in footprint counts in the beginning of the transcript was only subtle. However, the α group showed a significant increase in footprint counts (Figure 6F). Therefore, we concluded that only sequences with extremely low CSC can lead to a ribosome ramp. The ribosome ramp as seem in Figure 6C and G + CHX is indeed an artefact caused by the drug and is independent of codon composition.

Recently, the DEAD-box protein Dhh1p was shown to function as a sensor of codon optimality that targeted mRNA for degradation (14). In a series of elegant experiments, Coller *et al.* showed that Dhh1p bound to ribosomes and modulated ribosome occupancy of mRNAs with low codon optimality (14). The authors proposed that when elongation was delayed, Dhh1p bound to the translating ribosomes, leading to mRNA decay. To prove that hypothesis, a gene with optimal codons (PGK1) was modified with a non-endogenous stretch of ten codons with exceptionally low CSCs (TTA ATA GCG CGG CGG CGG CGG CGG GCG ACG). This stretch was placed at increasing distances from the ATG. Interestingly, the half-life of the PGK1 mRNA inversely correlated with the distances at which the stretch was placed (i.e., mRNAs with non-optimal codons that are closer to ATG possess longer half-lives than RNAs in which these stretches are located more distant from the ATG) (14). We investigated whether this observation could be expanded to endogenous mRNAs. Using the CSC score from Coller's dataset, we summed the value of each one of the ten codons used in Coller's PGK1 construct and found a value of –1.28. To determine the frequency and the position of stretches of non-optimal codons in the yeast genome, we developed a program that was able to screen the sequence of a given gene and calculate the sum of CSCs for every 10 codons. We found 1702 stretches in 1208 genes with values equal to or lower than –1.28. To allow a comparison with the optimal codons, we followed the same strategy, but this time the threshold value was equal to or higher than +1.28. The distribution of non-optimal stretches was not random; they were concentrated close to the ATG. An opposite less obvious distribution was observed with the optimal codons (Figure 7A). Next, we created a new dataset in which we removed genes with more than one non-optimal stretch and with undetermined mRNA half-lives (11). The genes were organized according to the position of the stretch with non-optimal codons from the ATG (Figure 7B, non-optimal stretch shown in yellow). Next, the ribosome profiles of the same genes were analyzed (Supplementary Figure S8). Notably, we used ribosome profiling data obtained in the absence of cycloheximide prior to cell lysis (30,45). We aligned the non-optimal codon stretches and compared the average ribosome density from the 500 nucleotides before and after the stretch of non-optimal codons (Figure 7D, red line). Again, all analysis steps used for genes with non-optimal stretches were repeated with genes with optimal stretches as a control (Figure 7D, blue line). We compared the normalized ribosome profiles of genes containing a non-optimal stretch vs. an optimal stretch (Figure 7D, right axis). As expected, ribosome accumulation was observed at the beginning of the non-optimal stretch, whereas no accumulation was present in the genes with an optimal stretch (Figure 7D, see adjusted p-values on the right y-axis). However, the statistical analysis did not support the existence of other differences in the ribosome distribution between these two groups of genes. A similar pattern was observed with ribosome profiling from another dataset (Supplementary Figure S9A) (30). This observation is important since the model proposed by Coller *et al.* to explain the difference in mRNA half-lives for genes with non-optimal codons is based on the accumulation of ribosomes before the stalling point (14). Based on the data obtained with the modified versions of PGK1, genes with a non-optimal stretch of codons located closer to the ATG should have higher half-lives than genes in which the stretch is located farther away from the start codon. When we compared the mRNA half-life value with the distance of the non-optimal stretch of codons from the ATG, we did not observe any correlation (Figure 7C and E). As expected, the same absence of correlation was also observed when the control group (genes with optimal stretches of codons) was analyzed (Supplementary Figure S9B). The presence of a non-optimal stretch affects the half-life of the mRNA (Figure 7F), but a direct correlation between the position of the non-optimal stretch and a reduction in the mRNA half-life was not observed in our analysis (Figure 7E). One possible explanation for the different conclusions obtained with the reporter system and our analysis may reside in the use of PGK1 as the reporter sequence. PGK1 is a highly expressed protein that is formed mainly by neutral or optimal codons. It belongs to cluster 1 of Figure 4 and has the 15th highest CSCg value in the yeast genome. We observed that the lowest CSC value in a window of 10 residues for this class was -0.055 (Supplementary Figure S10). Therefore, in this context, the occurrence of a stretch with –1.28 can be considered an aberration. The effect of the insertion of a non-optimal codon stretch in an optimized gene, such as PGK1, may not reflect the situation with naturally occurring sequences. In fact, most yeast genes are formed by non-optimal and neutral codons. For instance, all sequences used in Figure 7B and F belonged to cluster 3 of Figure 4B. In this context, the same stretch of non-optimal codons tested with PGK1 presented little impact on the overall mRNA stability, and the position-specific effect observed with the reporter was completely lost.

## CONCLUSIONS

Using CSCs for each of the 61 codons and the CSCg values calculated for each yeast gene, we elucidated important features of the yeast genome, including the following: (i) some mRNAs concentrate optimized and non-optimized codons, and this organization is shaped by evolution; (ii) genes with optimal codons can be found in highly expressed genes not only under standard growth conditions but also under stress conditions and (iii) stretches of non-optimal codons tend to be located closer to the ATG. The presence of these sequences can slow ribosome movement, but genes containing non-optimal stretches closer to the ATG do not necessarily have higher mRNA half-lives.

## DATA AVAILABILITY

The algorithms used in this work are available in the GitHub repository (https://github.com/RodolfoCarneiro/CodonOptmality/tree/master).

## Supplementary Material

Supplementary Data

## References

[1] Parker R. RNA degradation in Saccharomyces cerevisae. Genetics. 2012; 191:671–702. 22785621 10.1534/genetics.111.137265 PMC3389967

[2] Neymotin B., Ettorre V., Gresham D. Multiple transcript properties related to translation affect mRNA degradation rates in Saccharomyces cerevisiae. G3 (Bethesda). 2016; 6:3475–3483. 27633789 10.1534/g3.116.032276 PMC5100846

[3] Cheng J., Maier K.C., Avsec Ž., Rus P., Gagneur J. Cis-regulatory elements explain most of the mRNA stability variation across genes in yeast. RNA. 2017; 23:1648–1659. 28802259 10.1261/rna.062224.117 PMC5648033

[4] Hanson G., Coller J. Codon optimality, bias and usage in translation and mRNA decay. Nat. Rev. Mol. Cell Biol. 2018; 19:20–30. 29018283 10.1038/nrm.2017.91 PMC6594389

[5] Sharp P.M., Li W.H. The codon adaptation index—a measure of directional synonymous codon usage bias, and its potential applications. Nucleic Acids Res. 1987; 15:1281–1295. 3547335 10.1093/nar/15.3.1281 PMC340524

[6] Pechmann S., Frydman J. Evolutionary conservation of codon optimality reveals hidden signatures of cotranslational folding. Nat. Struct. Mol. Biol. 2012; 20:237–243. 23262490 10.1038/nsmb.2466 PMC3565066

[7] Rocha E.P.C. Codon usage bias from tRNA’s point of view: redundancy, specialization, and efficient decoding for translation optimization. Genome Res. 2004; 14:2279–2286. 15479947 10.1101/gr.2896904 PMC525687

[8] Dong H., Nilsson L., Kurland C.G. Co-variation of tRNA abundance and codon usage in Escherichia coli at different growth rates. J. Mol. Biol. 1996; 260:649–663. 8709146 10.1006/jmbi.1996.0428

[9] Reis dos M., Savva R., Wernisch L. Solving the riddle of codon usage preferences: a test for translational selection. Nucleic Acids Res. 2004; 32:5036–5044. 15448185 10.1093/nar/gkh834 PMC521650

[10] Herrick D., Parker R., Jacobson A. Identification and comparison of stable and unstable mRNAs in Saccharomyces cerevisiae. Mol. Cell. Biol. 1990; 10:2269–2284. 2183028 10.1128/mcb.10.5.2269-2284.1990 PMC360574

[11] Presnyak V., Alhusaini N., Chen Y.-H., Martin S., Morris N., Kline N., Olson S., Weinberg D., Baker K.E., Graveley B.R. et al. Codon optimality is a major determinant of mRNA stability. Cell. 2015; 160:1111–1124. 25768907 10.1016/j.cell.2015.02.029 PMC4359748

[12] Baudrimont A., Voegeli S., Viloria E.C., Stritt F., Lenon M., Wada T., Jaquet V., Becskei A. Multiplexed gene control reveals rapid mRNA turnover. Sci. Adv. 2017; 3:e1700006. 28706991 10.1126/sciadv.1700006 PMC5507631

[13] Harigaya Y., Parker R. Analysis of the association between codon optimality and mRNA stability in Schizosaccharomyces pombe. BMC Genomics. 2016; 17:129–116. 27825301 10.1186/s12864-016-3237-6 PMC5101800

[14] Radhakrishnan A., Chen Y.-H., Martin S., Alhusaini N., Green R., Coller J. The DEAD-Box protein dhh1p couples mRNA decay and translation by monitoring codon optimality. Cell. 2016; 167:122–132. 27641505 10.1016/j.cell.2016.08.053 PMC5635654

[15] Holstege F.C., Jennings E.G., Wyrick J.J., Lee T.I., Hengartner C.J., Green M.R., Golub T.R., Lander E.S., Young R.A. Dissecting the regulatory circuitry of a eukaryotic genome. Cell. 1998; 95:717–728. 9845373 10.1016/s0092-8674(00)81641-4

[16] Wang Y., Wang Y., Liu C.L., Storey J.D., Liu C.L., Storey J.D., Brown P.O., Tibshirani R.J., Tibshirani R.J., Herschlag D. et al. Precision and functional specificity in mRNA decay. Proc. Natl. Acad. Sci. U.S.A. 2002; 99:5860–5865. 11972065 10.1073/pnas.092538799 PMC122867

[17] Grigull J., Grigull J., Mnaimneh S., Mnaimneh S., Pootoolal J., Robinson M.D., Pootoolal J., Robinson M.D., Hughes T.R., Hughes T.R. Genome-Wide analysis of mRNA stability using transcription inhibitors and microarrays reveals posttranscriptional control of ribosome biogenesis factors. Mol. Cell. Biol. 2004; 24:5534–5547. 15169913 10.1128/MCB.24.12.5534-5547.2004 PMC419893

[18] Geisberg J.V., Moqtaderi Z., Fan X., Ozsolak F., Struhl K. Global analysis of mRNA isoform Half-Lives reveals stabilizing and destabilizing elements in Yeast. Cell. 2014; 156:812–824. 24529382 10.1016/j.cell.2013.12.026 PMC3939777

[19] Munchel S.E., Shultzaberger R.K., Munchel S.E., Shultzaberger R.K., Weis K., Takizawa N., Takizawa N., Weis K. Dynamic profiling of mRNA turnover reveals gene-specific and system-wide regulation of mRNA decay. Mol. Biol. Cell. 2011; 22:2787–2795. 21680716 10.1091/mbc.E11-01-0028 PMC3145553

[20] Neymotin B., Athanasiadou R., Gresham D. Determination of in vivo RNA kinetics using RATE-seq. RNA. 2014; 20:1645–1652. 25161313 10.1261/rna.045104.114 PMC4174445

[21] Sun M., Sun M., Schwalb B., Schulz D., Pirkl N., Schwalb B., Etzold S., Schulz D., Maier K.C., Pirkl N. et al. Comparative dynamic transcriptome analysis (cDTA) reveals mutual feedback between mRNA synthesis and degradation. Genome Res. 2012; 22:1350–1359. 22466169 10.1101/gr.130161.111 PMC3396375

[22] Wada T., Becskei A. Impact of Methods on the Measurement of mRNA Turnover. Int. J. Mol. Sci. 2017; 18:2723. 10.3390/ijms18122723 PMC5751324 29244760

[23] Requião R.D., Fernandes L., de Souza H.J.A., Rossetto S., Domitrovic T., Palhano F.L. Protein charge distribution in proteomes and its impact on translation. PLoS Comput. Biol. 2017; 13:e1005549. 28531225 10.1371/journal.pcbi.1005549 PMC5460897

[24] Requião R.D., de Souza H.J.A., Rossetto S., Domitrovic T., Palhano F.L. Increased ribosome density associated to positively charged residues is evident in ribosome profiling experiments performed in the absence of translation inhibitors. RNA Biol. 2016; 13:561–568. 27064519 10.1080/15476286.2016.1172755 PMC4962802

[25] Ingolia N.T., Brar G.A., Rouskin S., McGeachy A.M., Weissman J.S. The ribosome profiling strategy for monitoring translation in vivo by deep sequencing of ribosome-protected mRNA fragments. Nat. Protoc. 2012; 7:1534–1550. 22836135 10.1038/nprot.2012.086 PMC3535016

[26] Boël G., Letso R., Neely H., Price W.N., Wong K.-H., Su M., Luff J.D., Valecha M., Everett J.K., Acton T.B. et al. Codon influence on protein expression in E. coli correlates with mRNA levels. Nature. 2016; 529:358–363. 26760206 10.1038/nature16509 PMC5054687

[27] Weinberg D.E., Shah P., Eichhorn S.W., Hussmann J.A., Plotkin J.B., Bartel D.P. Improved Ribosome-Footprint and mRNA measurements provide insights into dynamics and regulation of Yeast translation. Cell Rep. 2016; 14:1787–1799. 26876183 10.1016/j.celrep.2016.01.043 PMC4767672

[28] Tuller T., Carmi A., Vestsigian K., Navon S., Dorfan Y., Zaborske J., Pan T., Dahan O., Furman I., Pilpel Y. An evolutionarily conserved mechanism for controlling the efficiency of protein translation. Cell. 2010; 141:344–354. 20403328 10.1016/j.cell.2010.03.031

[29] Ingolia N.T., Ghaemmaghami S., Newman J.R.S., Weissman J.S. Genome-Wide analysis in vivo of translation with nucleotide resolution using ribosome profiling. Science. 2009; 324:218–223. 19213877 10.1126/science.1168978 PMC2746483

[30] Gerashchenko M.V., Gladyshev V.N. Translation inhibitors cause abnormalities in ribosome profiling experiments. Nucleic Acids Res. 2014; 42:e134. 25056308 10.1093/nar/gku671 PMC4176156

[31] Hussmann J.A., Patchett S., Johnson A., Sawyer S., Press W.H. Understanding biases in ribosome profiling experiments reveals signatures of translation dynamics in Yeast. PLoS Genet. 2015; 11:e1005732. 26656907 10.1371/journal.pgen.1005732 PMC4684354

[32] Fang H., Huang Y.-F., Radhakrishnan A., Siepel A., Lyon G.J., Schatz M.C. Scikit-ribo enables accurate estimation and robust modeling of translation dynamics at codon resolution. Cell Syst. 2018; 6:180–191. 29361467 10.1016/j.cels.2017.12.007 PMC5832574

[33] Gardin J., Yeasmin R., Yurovsky A., Cai Y., Skiena S., Futcher B. Measurement of average decoding rates of the 61 sense codons in vivo. Elife. 2014; 3:198. 10.7554/eLife.03735 PMC4371865 25347064

[34] Hanson G., Alhusaini N., Morris N., Sweet T., Coller J. Translation elongation and mRNA stability are coupled through the ribosomal A-site. RNA. 2018; 24:1377–1389. 29997263 10.1261/rna.066787.118 PMC6140462

[35] Lahtvee P.-J., Sánchez B.J., Smialowska A., Kasvandik S., Elsemman I.E., Gatto F., Nielsen J. Absolute quantification of protein and mRNA abundances demonstrate variability in gene-specific translation efficiency in yeast. Cell Syst. 2017; 4:495–504. 28365149 10.1016/j.cels.2017.03.003

[36] Yassour M., Kaplan T., Fraser H.B., Levin J.Z., Pfiffner J., Adiconis X., Schroth G., Luo S., Khrebtukova I., Gnirke A. et al. Ab initio construction of a eukaryotic transcriptome by massively parallel mRNA sequencing. Proc. Natl. Acad. Sci. U.S.A. 2009; 106:3264–3269. 19208812 10.1073/pnas.0812841106 PMC2638735

[37] Ikemura T. Correlation between the abundance of Escherichia coli transfer RNAs and the occurrence of the respective codons in its protein genes. J. Mol. Biol. 1981; 146:1–21. 6167728 10.1016/0022-2836(81)90363-6

[38] Ikemura T. Correlation between the abundance of yeast transfer RNAs and the occurrence of the respective codons in protein genes. Differences in synonymous codon choice patterns of yeast and Escherichia coli with reference to the abundance of isoaccepting transfer RNAs. J. Mol. Biol. 1982; 158:573–597. 6750137 10.1016/0022-2836(82)90250-9

[39] Heyer E.E., Moore M.J. Redefining the translational status of 80S monosomes. Cell. 2016; 164:757–769. 26871635 10.1016/j.cell.2016.01.003

[40] Kulak N.A., Pichler G., Paron I., Nagaraj N., Mann M. Minimal, encapsulated proteomic-sample processing applied to copy-number estimation in eukaryotic cells. Nat. Methods. 2014; 11:319–324. 24487582 10.1038/nmeth.2834

[41] Ho B., Baryshnikova A., Brown G.W. Unification of protein abundance datasets yields a quantitative saccharomyces cerevisiae proteome. Cell Syst. 2018; 6:192–205. 29361465 10.1016/j.cels.2017.12.004

[42] Leandro M.J., Fonseca C.S., GonÃ alves P. Hexose and pentose transport in ascomycetous yeasts: an overview. FEMS Yeast Res. 2009; 9:511–525. 19459982 10.1111/j.1567-1364.2009.00509.x

[43] Breker M., Gymrek M., Schuldiner M. A novel single-cell screening platform reveals proteome plasticity during yeast stress responses. J. Cell Biol. 2013; 200:839–850. 23509072 10.1083/jcb.201301120 PMC3601363

[44] Tuller T., Veksler-Lublinsky I., Gazit N., Kupiec M., Ruppin E., Ziv-Ukelson M. Composite effects of gene determinants on the translation speed and density of ribosomes. Genome Biol. 2011; 12:R110. 22050731 10.1186/gb-2011-12-11-r110 PMC3334596

[45] Pop C., Rouskin S., Ingolia N.T., Han L., Phizicky E.M., Weissman J.S., Koller D. Causal signals between codon bias, mRNA structure, and the efficiency of translation and elongation. Mol. Syst. Biol. 2014; 10:770. 25538139 10.15252/msb.20145524 PMC4300493

