# Supplementary material for: Codon stabilization coefficient as a metric to gain insights into mRNA stability and codon bias and their relationships with translation
Source: Nucleic Acids Res. 2019 Jan 30;47(5):2216–28. doi: 10.1093/nar/gkz033 (PMC6412131; doi:10.1093/nar/gkz033)
Supplement: Supplementary Data [file gkz033_supplemental_files.zip › Carneiro et al Support Material_R2.pdf]

Support Material for:

Codon stabilization coefficient as a metric to gain insights into mRNA stability and codon bias and their relationships with translation.

Rodolfo L. Carneiro[1], Rodrigo D. Requião[1], Silvana Rossetto[2], Tatiana Domitrovic[3]* and Fernando L. Palhano[1]*
[1] Programa de Biologia Estrutural, Instituto de Bioquímica Médica Leopoldo de Meis, Universidade Federal do Rio de Janeiro, Rio de Janeiro, RJ, 21941-902, Brazil.
[2] Departamento de Ciência da Computação, Universidade Federal do Rio de Janeiro, Rio de Janeiro, RJ, 21941-902, Brazil.
[3] Departamento de Virologia, Instituto de Microbiologia Paulo de Góes, Universidade Federal do Rio de Janeiro, Rio de Janeiro, 21941-902, Brazil.

* To whom correspondence should be addressed: Tel.: +55 21 3938-6761; Email: palhano@bioqmed.ufrj.br or domitrovic@micro.ufrj.br

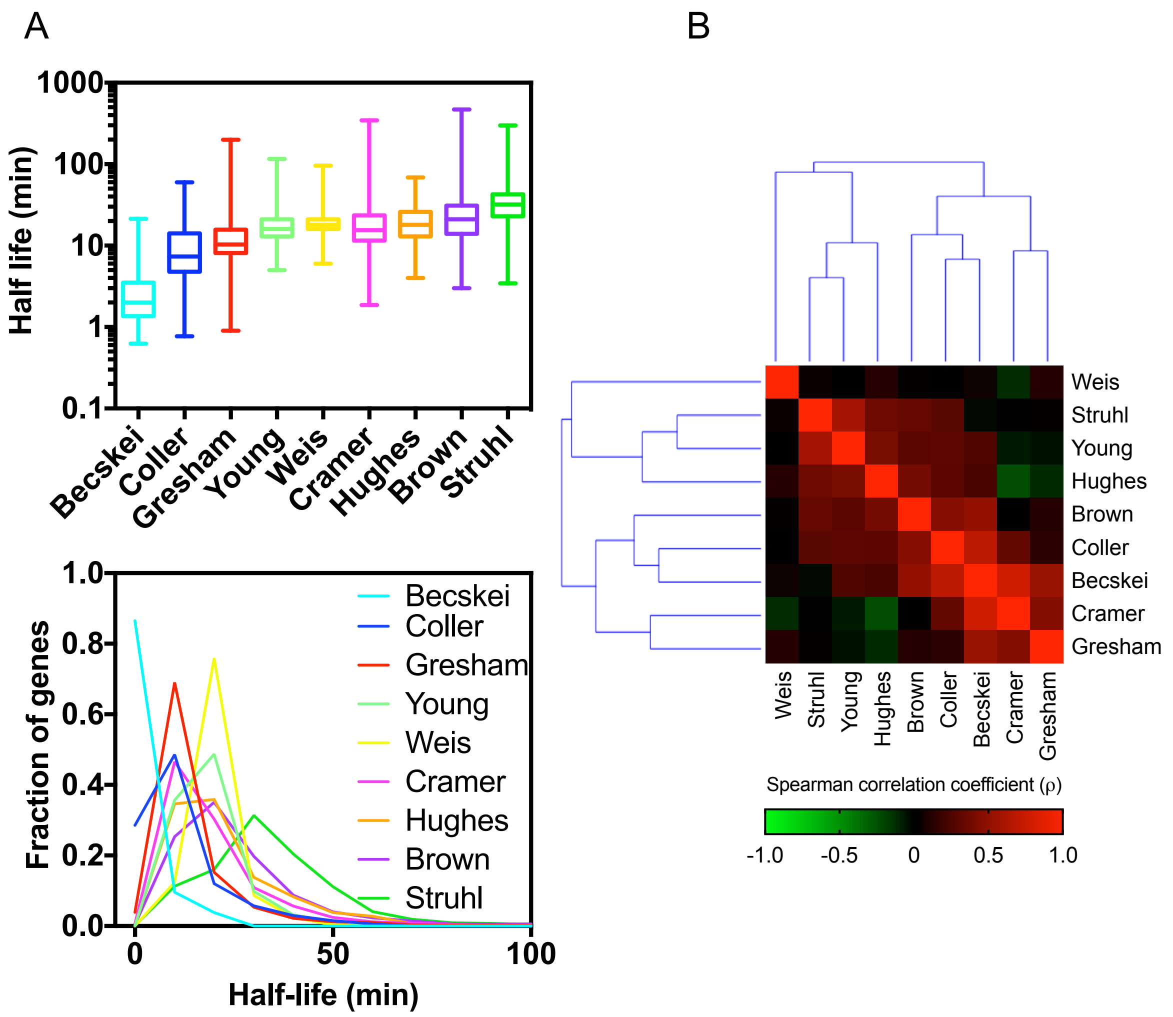

Figure S1. Comparison of *S. cerevisiae* mRNA half-lives generated by different studies. A. Description of the experiments analyzed in this work, namely, Young, Brown, Hughes, Coller, Struhl, Weis, Gresham, Cramer and Becskei. B. Upper and lower panel show the average mRNA half-life and the frequency distribution of mRNA half-life measurements, respectively. C. Spearman correlation between the datasets. The heat map shows the correlation coefficients (r) ranging from r -0.25 (negative correlation, green panels) to 1.00 (positive correlation, red panels). The raw data, sample size, p values, uncorrected critical ρ and Spearman's correlation coefficient (ρ) are present in Supplemental Table S1.

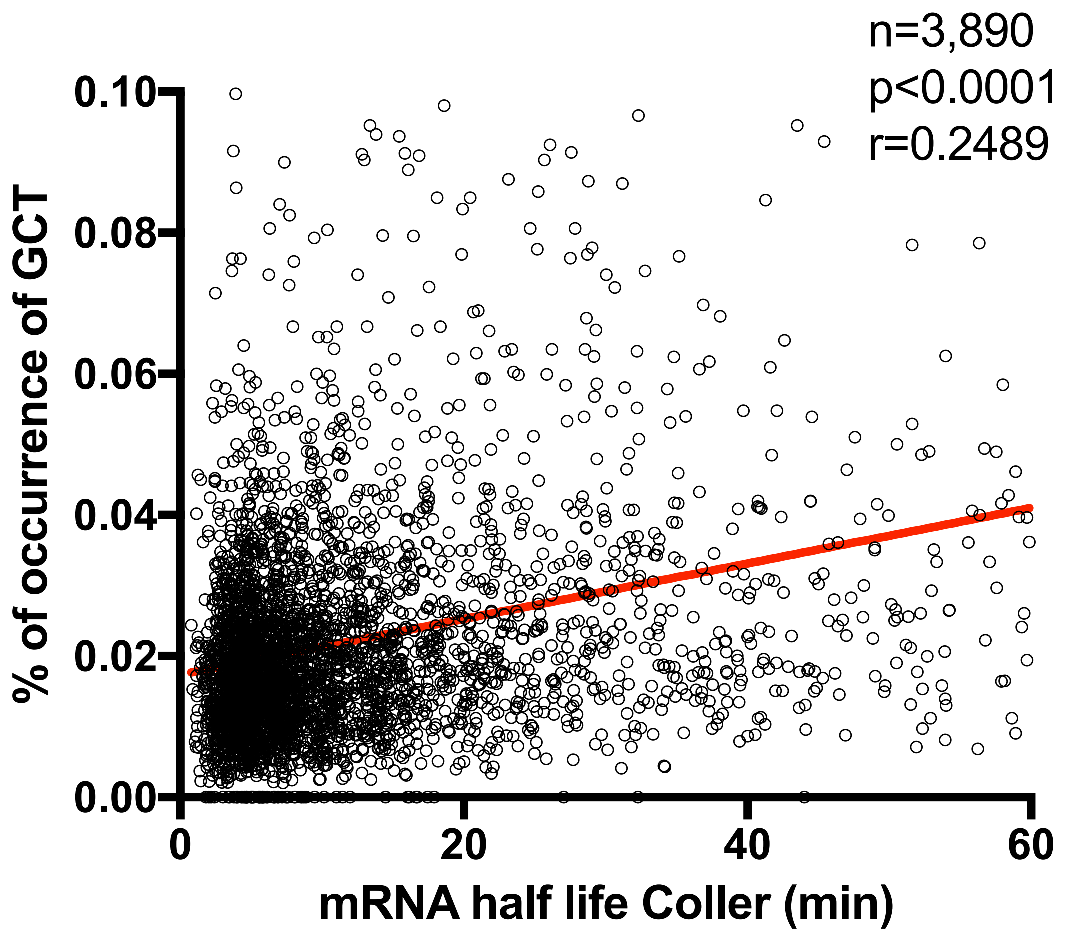

Figure S2. Calculation of codon stabilization coefficient. The CSC for each of the 61 amino-acid coding codons equals the correlation coefficient (r) derived from Pearson's correlation between the frequency of occurrence of each codon in the transcriptome and the mRNA half-lives values. As an example, we show the plot used to derive the CSC for the codon GTC using Coller's mRNA half-life measurements.

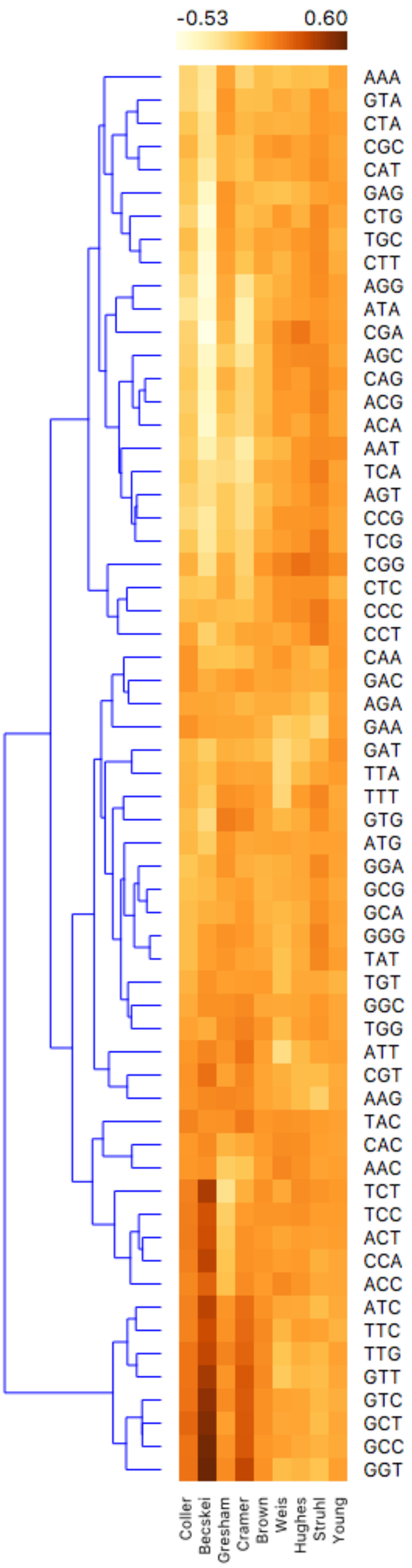

Figure S3. The codon stabilization coefficient for each 61 amino-acid coding codons calculated from the 9 datasets used in this work (Table 1). The raw data, sample size, p values, uncorrected critical ρ and Spearman's correlation coefficient (ρ) are present in Supplemental Table S2.

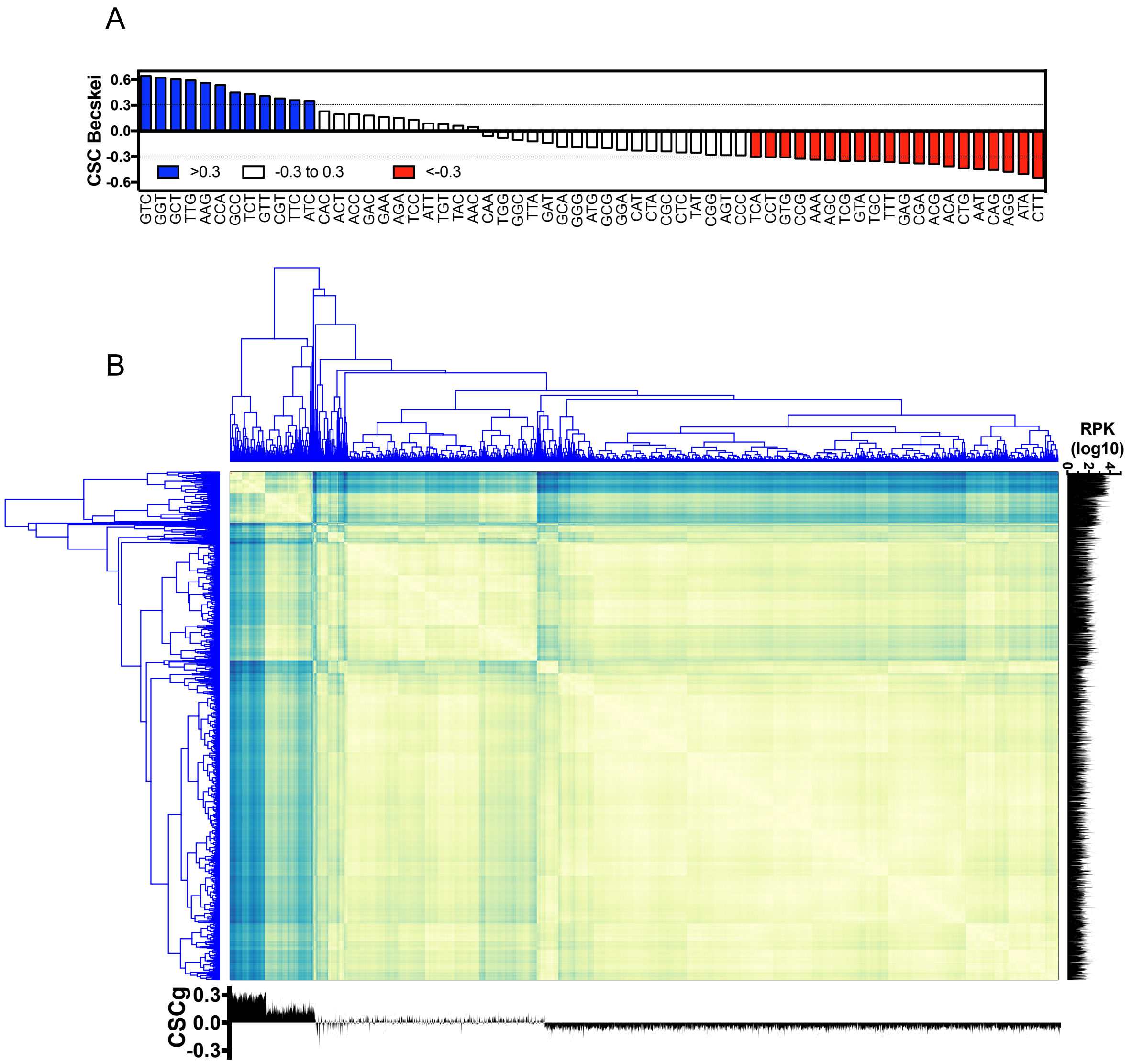

Figure S4. Evaluation of mRNA codon composition and gene expression using the CSCg and the CSC. A. The 61 codons were divided into three groups according to their CSC values (Becskei's dataset): optimal codons are shown in blue (CSC > 0.3), neutral codons are shown in white (0.3 > CSC > -0.3) and non-optimal codons are shown in red (CSC < -0.3). B. Distance matrix created from a gene level clustering based on the attributes described in panel A. Four clusters were found. The number of reads associated with ribosomes is represented on the right (RPK), and the CSCg for each gene is represented on the bottom.

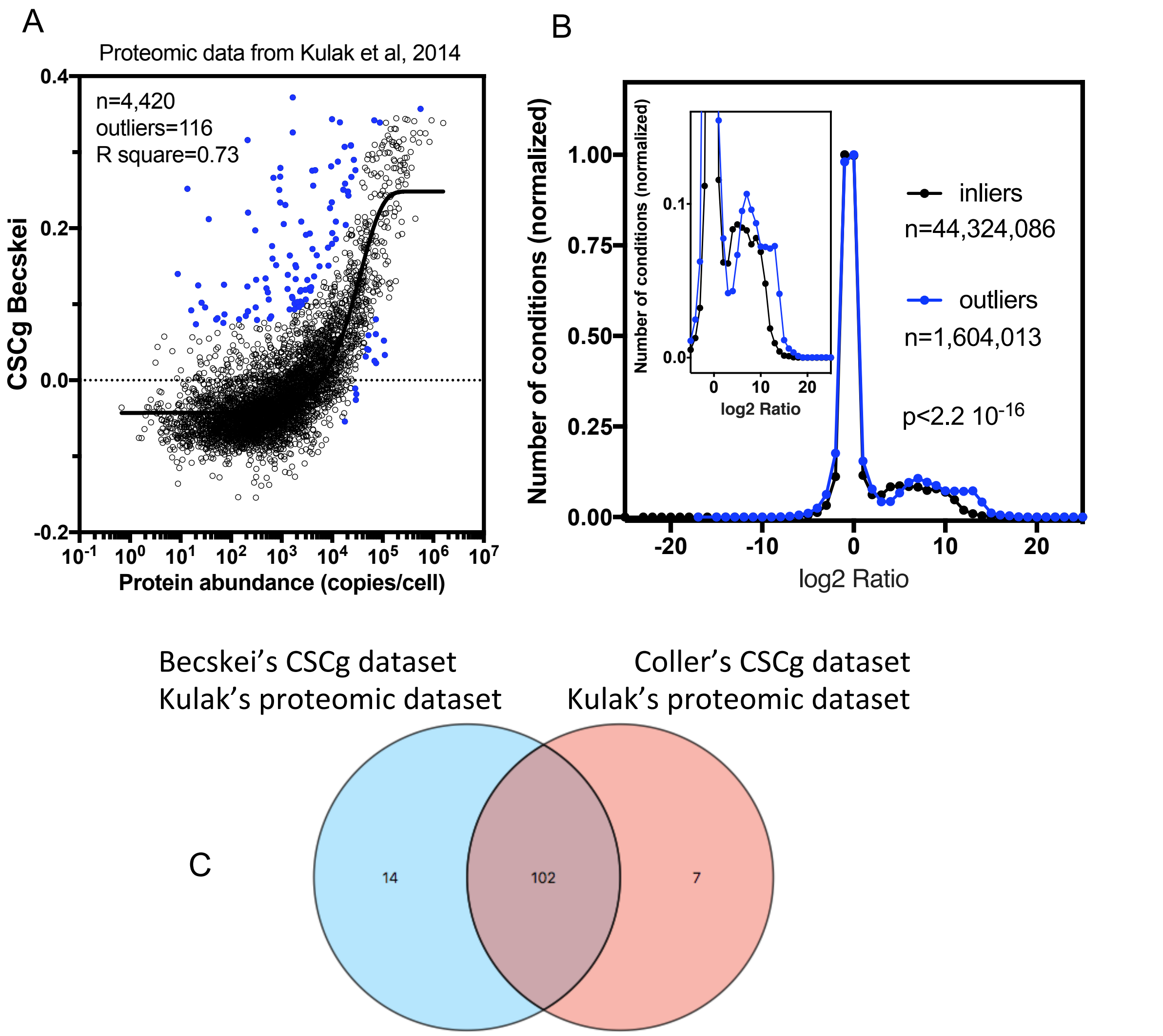

Figure S5. Correlation between CSCg and the copy number of proteins in *S. cerevisiae*. A. The best-fit curve was used to calculate the correlation among CSCg (Becskei's dataset) and protein abundance (Kulak's dataset). Outliers are highlighted in blue. B. Plot showing the distribution of genes according to their expression regulation at 400 different experimental conditions (data obtained from SGD). The inliers genes are shown in black line, and outliers subset of genes are shown in blue lines. The inset shows the overexpressed population on a different scale. Note that outliers presented higher levels of induction and overexpressed in more conditions than the inliers group. The p-value was calculated by Kolmogorov-Smirnov test (Table S5A) C. The same analyze descript in panel A was performed by comparison of other dataset namely, CSCg from Coller's dataset vs protein abundance from Kulak's dataset. The Venn diagram of outliers identified in these two analyses showed 102 genes in common.

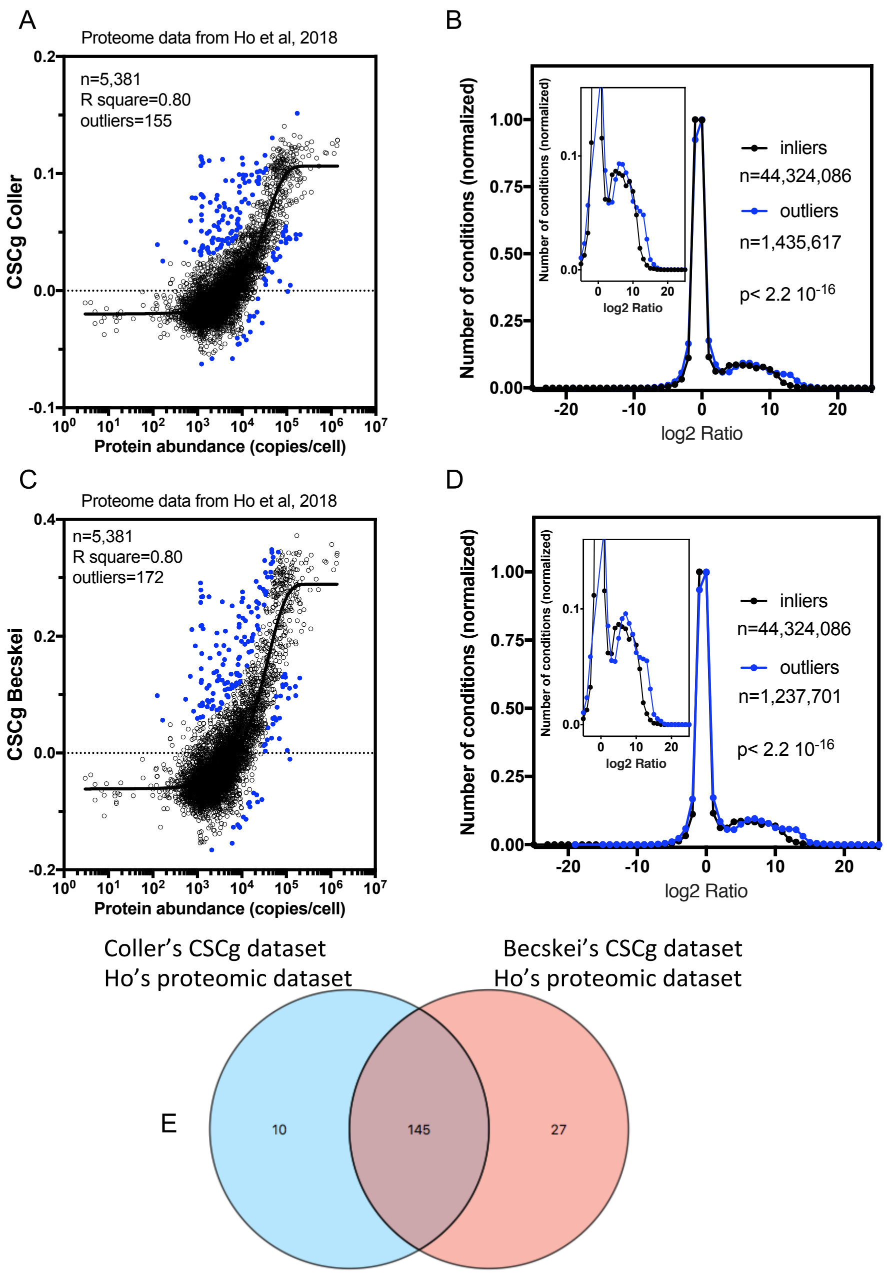

Figure S6. Correlation between CSCg and the copy number of proteins in *S. cerevisiae*. The best-fit curve was used to calculate the correlation among CSCg (Coller's dataset; A or Becskei's dataset; C) and protein abundance (Ho's dataset; A and C). Outliers are highlighted in blue. B and D. Plot showing the distribution of genes according to their expression regulation at 400 different experimental conditions (data obtained from SGD). The inliers genes are shown in black line, and outliers subset of genes are shown in blue lines. The inset shows the overexpressed population on a different scale. The p-value was calculated by Kolmogorov-Smirnov test (Table S5A) E. The Venn diagram of outliers identified in these two analyses showed 145 genes in common.

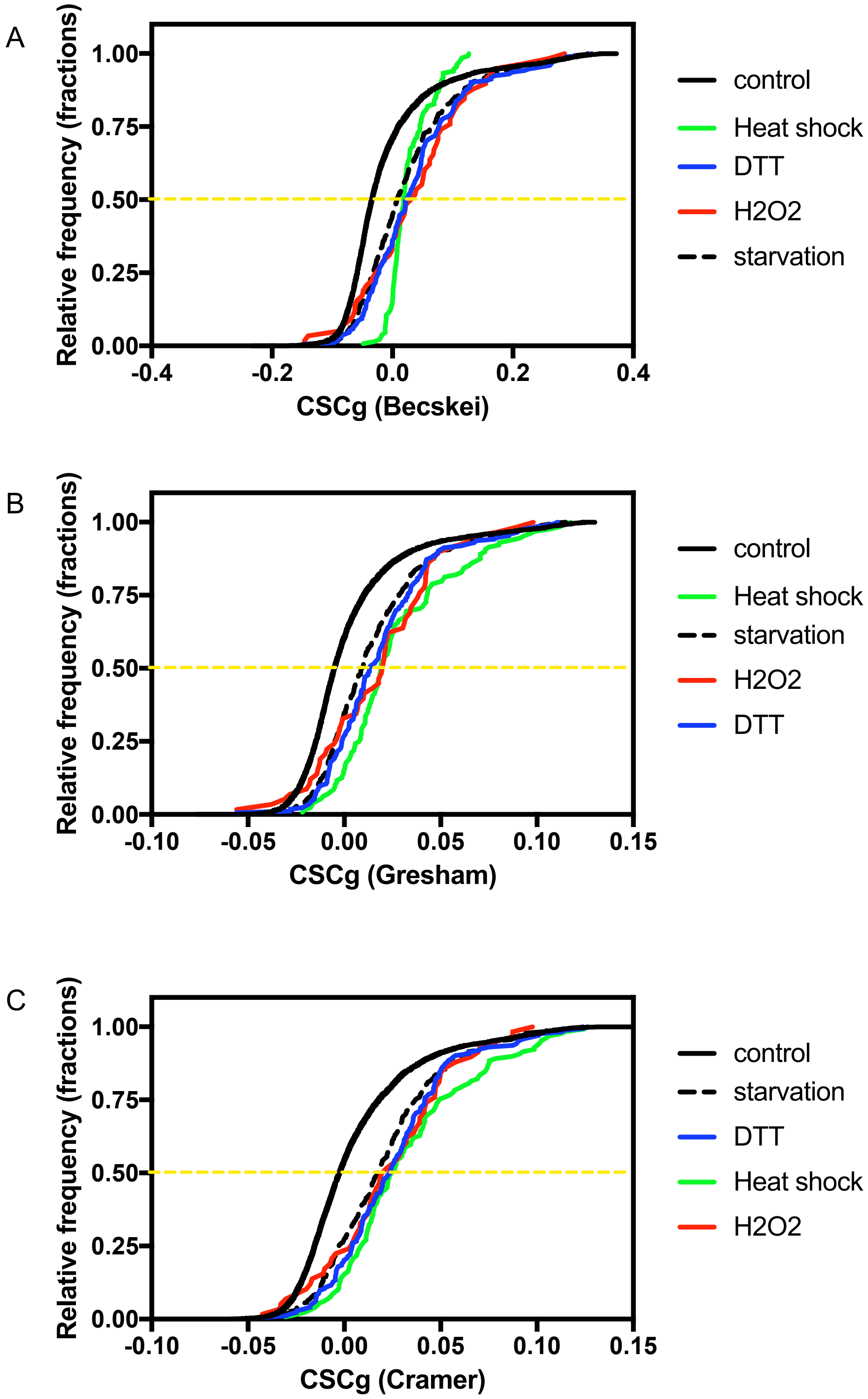

Figure S7. Stress genes induced genes different possess optimized CSC. The CSCg was calculated for a subset of proteins that were up-regulated by different stress conditions. Proteins with induction level more than 1.5 times were selected. Cumulative distribution of the CSCg (calculated from Becskei's (A), Gresham's (B) or Cramer's (C) datasets) of genes coding for the proteins that were induced more than 1.5 fold at different stresses was compared to all genes (control). The p-value calculated by Kolmogorov-Smirnov test was <0.0001 for each stress condition analyzed vs. the control (See statistics details on Table S5B).

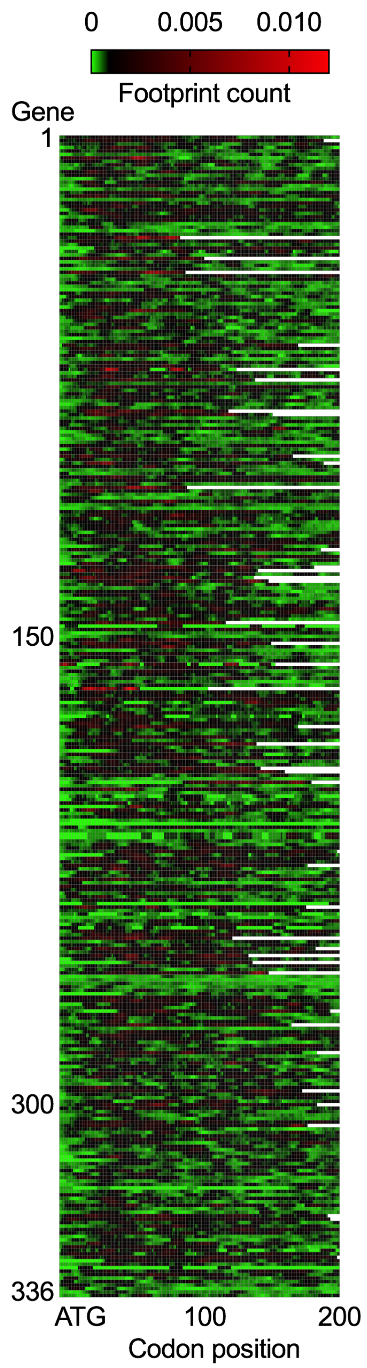

Figure S8. The heat map depicts the normalized footprint reads from ribosome profiling analyzes of the genes presented at Figure 7B and 7C. The heat map ranging from 0.005 to 0.010 is used to present the number of reads at each position normalized by the total number of reads in each gene.

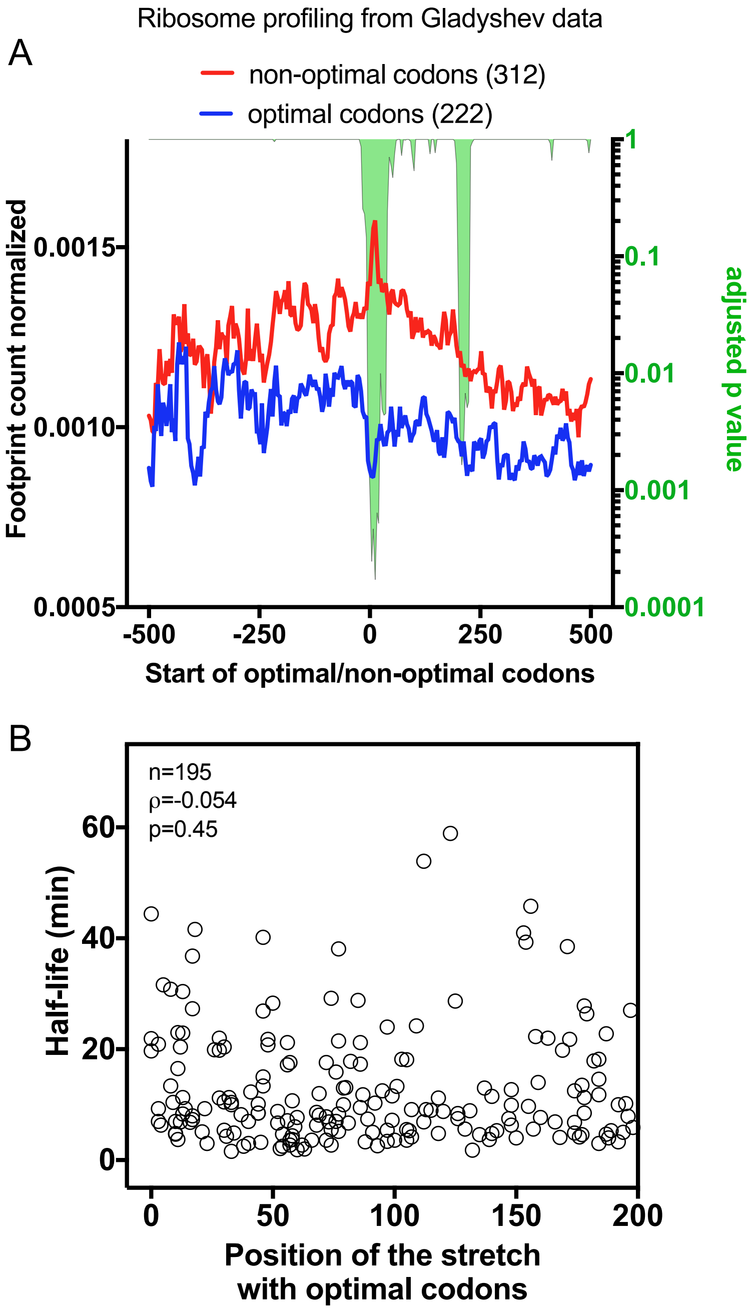

Figure S9. Occurrence and position of non-optimal/optimal codons stretch in yeast mRNAs and its impact on translation. A. Average ribosome footprint density (45) of the genes presented on panel 7B from the stretch of non-optimal codons (position 0). The number of reads at each position was normalized by the total number of reads at a window of 500 nucleotides before and after the stretch. Genes with optimal stretches CSC (≥1.28) (blue line) were used as controls group. The right y-axis represents the adjusted p-value (green area) calculated for each nucleotide position by multiple t-tests using the Holm-Sidak method. B. The mRNA half-life values (12) were plotted according to the position of the optimal stretch codons (CSC ≥1.28) from the ATG. No correlation was found between optimal codon position and mRNA half-life.

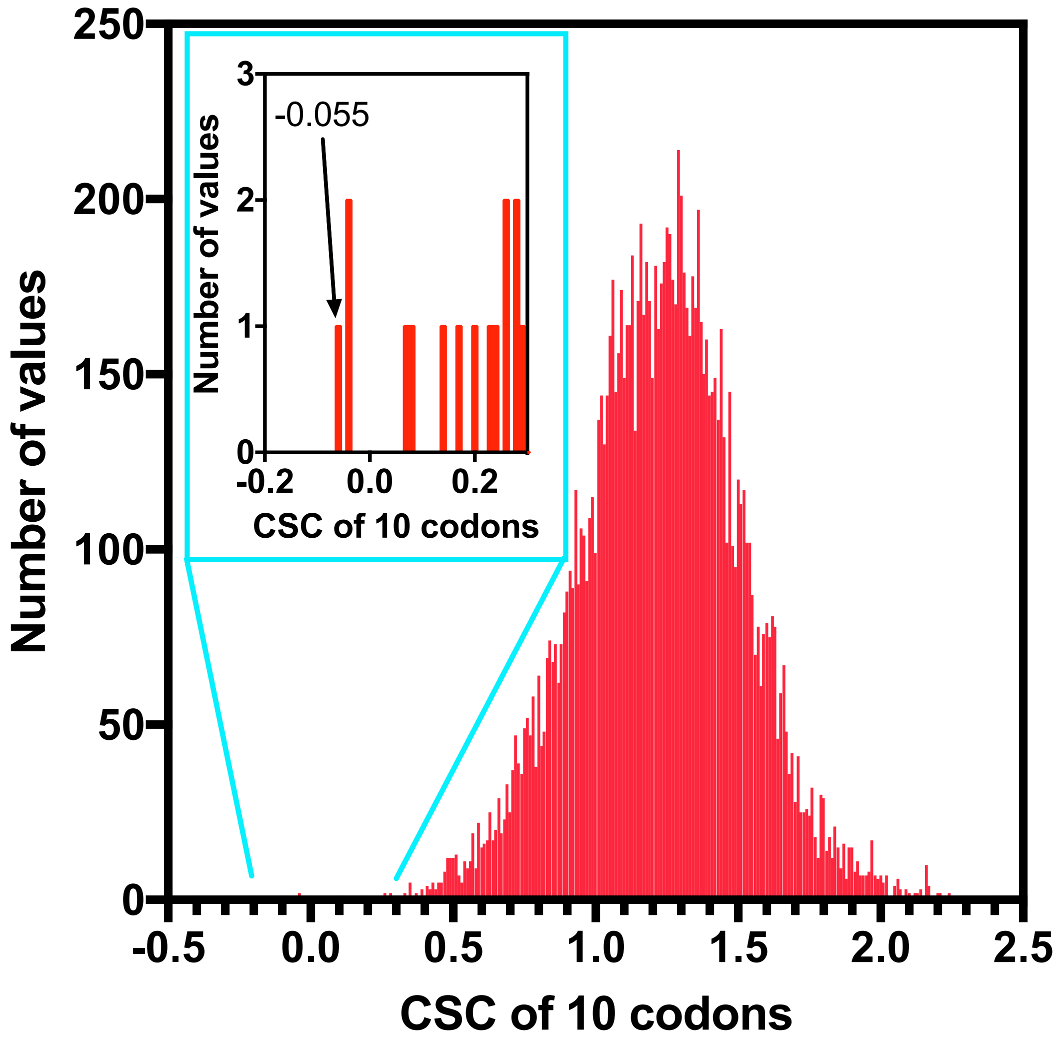

Figure S10. Frequency of distribution of the sum of CSCs of every 10 codons of the top 50 genes with the highest CSCg in yeast. The inset shows that the lowest score found in those genes has a value of -0.055.
